# State-of-the-Art Research on Chemiresistive Gas Sensors in Korea: Emphasis on the Achievements of the Research Labs of Professors Hyoun Woo Kim and Sang Sub Kim

**DOI:** 10.3390/s22010061

**Published:** 2021-12-23

**Authors:** Sachin Navale, Ali Mirzaei, Sanjit Manohar Majhi, Hyoun Woo Kim, Sang Sub Kim

**Affiliations:** 1 Division of Materials Science and Engineering, Hanyang University, Seoul 04763, Korea; stnavale2@yahoo.com (S.N.); sanjeetmjh@gmail.com (S.M.M.); 2 The Research Institute of Industrial Science, Hanyang University, Seoul 04763, Korea; 3 Department of Materials Science and Engineering, Inha University, Incheon 22212, Korea; 4 Department of Materials Science and Engineering, Shiraz University of Technology, Shiraz 715557-13876, Iran; mirzaei@sutech.ac.ir

**Keywords:** gas sensor, core–shell sensors, self-heated sensors, flexible sensors, irradiation sensors, sensing mechanism

## Abstract

This review presents the results of cutting-edge research on chemiresistive gas sensors in Korea with a focus on the research activities of the laboratories of Professors Sang Sub Kim and Hyoun Woo Kim. The advances in the synthesis techniques and various strategies to enhance the gas-sensing performances of metal-oxide-, sulfide-, and polymer-based nanomaterials are described. In particular, the gas-sensing characteristics of different types of sensors reported in recent years, including core–shell, self-heated, irradiated, flexible, Si-based, glass, and metal–organic framework sensors, have been reviewed. The most crucial achievements include the optimization of shell thickness in core–shell gas sensors, decrease in applied voltage in self-heated gas sensors to less than 5 V, optimization of irradiation dose to achieve the highest response to gases, and the design of selective and highly flexible gas sensors-based WS_2_ nanosheets. The underlying sensing mechanisms are discussed in detail. In summary, this review provides an overview of the chemiresistive gas-sensing research activities led by the corresponding authors of this manuscript.

## 1. Introduction: Overview of the Oxide-Based Gas Sensors

Atmospheric pollution typically comprises particulate matter, ozone (O_3_), sulfur dioxide (SO_2_), nitrogen dioxide (NO_2_), and carbon monoxide (CO) [1]. Furthermore, benzene (C_6_H_6_), toluene (C_7_H_8_), and xylene (C_8_H_10_) (BTX) gases are also generally present in pollutant air. It is considered that 92% of the world’s population lives in areas with poor air quality, which can cause premature death [2]. Pollution is the most important cause of premature death and illness worldwide, and it accounts for 16% of global death [3]. As a result, air pollution is currently an active area of research. Although the human olfactory system is considered to be the least significant of the human senses, it is critical to human lives because it alerts us to the potential risks pollutant air. In addition, it is the only active sense during sleep. When a gas has an extremely low concentration or no odor, the human olfactory system cannot sense it. As a result, sensitive devices are required to detect toxic and hazardous gases in the environment [4,5]. Gas chromatography-mass spectrometry [6], flame ionization detectors [7], and Fourier-transform infrared spectrometry [8] have been traditionally used for the recognition of toxic/hazardous gases. However, most of these systems are large in size, costly, and require trained operators. As the instruments are not portable because of their large sizes, their applications in different areas are limited [9]. Additionally, these instruments cannot be incorporated into small smart electronic devices such as smartphones. Thus, sensitive and reliable sensing devices with small sizes are needed to be incorporated in portable devices.

Table 1 presents the historical milestones of semiconductor-based chemiresistive gas sensors [10,11,12,13,14,15,16,17]. The idea of utilizing metal oxides as gas-detecting materials dates back to 1954 when Heiland applied zinc oxide (ZnO) as a sensing material [10], extending the original idea of Brattain and Barteen describing the change in the composition of the surrounding atmosphere on the electrical conductivity of Ge-based electronic devices [11]. Motivated by the extensive gas explosions in Japan, Seiyama (1962) invented a simple resistive-based gas sensor using ZnO thin films for sensing propane, which afforded a response approximately 100 times higher than the thermal conductivity detector utilized at the time [12]. In 1968, Taguchi commercialized a simple gas sensor using semiconducting tin oxide (SnO_2_) to detect low concentrations of combustible/reducing gases [13]. 

To date, a variety of gas sensors have been utilized to detect toxic and hazardous gases. Among these, metal–oxide semiconductor (MOS) gas sensors, whose operation is based on the change in the resistance upon exposure to target gases, have attracted significant attention owing to their low cost, low toxicity, simple fabrication route, robustness, high stability, fast response/recovery times, and sensitivity to detect a wide range of target gases [18,19,20,21,22,23,24,25,26,27,28,29,30,31,32,33,34,35,36,37,38,39,40,41,42,43,44,45,46,47,48,49,50,51,52,53,54,55,56,57,58,59,60,61,62,63]. Notably, in addition to MOS, materials based on conducting polymers and graphene have been employed for the realization of resistive-gas sensors [18]. However, their performances, particularly in the pristine form, is significantly inferior to those of the MOS-based sensors. The main characteristics of a resistive-gas sensor are its response, selectivity, response time, stability, and sensing temperature. In other words, a good gas sensor should (i) afford a high response to the target gas, (ii) show no or low response to interfering gases, (iii) show high stability over a long time, and (iv) be used at low or room temperatures to minimize energy consumption [18]. Thus, various synthesis approaches or strategies have been used to overcome the limitations of this type of gas sensors: namely, poor selectivity and high operating temperature. A comparison of the most commonly used pristine metal oxides, with different surface morphologies, as a sensing layer in chemiresistive-type of gas sensors is presented in Table 2 [19,20,21,22,23,24,25,26,27,28,29,30,31,32,33,34,35,36,37,38,39,40,41,42,43,44,45,46,47,48,49,50,51,52,53,54,55,56,57,58,59,60,61,62,63].

**Scheme 1:**
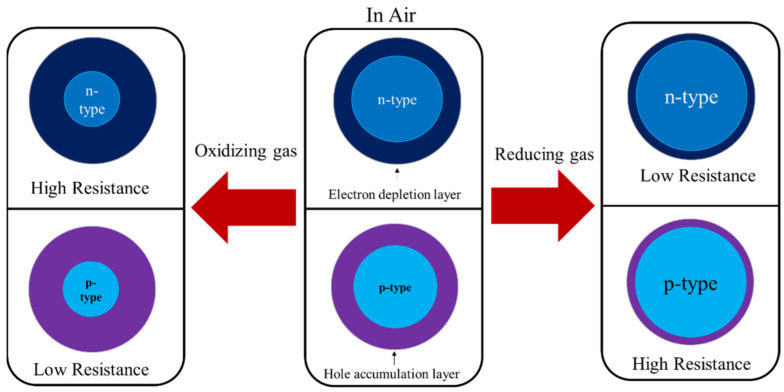
General gas-sensing mechanism of *n-* and *p-type* MOS-based chemiresistors in oxidizing and reducing gas atmosphere.

The general gas-sensing mechanism of resistive-based gas sensors is mainly based on the resistance variations of the sensing materials in the presence of target gases [19,20,21,22,23,24,25,26,27,28,29,30,31,32,33,34,35,36,37,38,39,40,41,42,43,44,45,46,47,48,49,50,51,52,53,54,55,56,57,58,59,60,61,62,63]. A scheme of the general detection mechanism of resistive-based *n-type* and *p-type* MOSs sensors, in the presence of oxidizing/reducing gases, is presented in Scheme 1. As shown in Scheme 1, in air, an electron depletion layer (EDL) and a hole accumulation layer (HAL) forms on the surfaces of *n-type* and *p-type* MOSs, respectively, due to the abstraction of electrons by adsorbed oxygen species on the surface of the gas sensor. Considering an *n-type* gas sensor, when it is exposed to reducing gases, due to the reaction between the gas and adsorbed oxygen species, the electrons will be released and come back to the sensor surface, leading to a decrease in the width of the EDL and increase in the sensor resistance. Upon exposure to oxidizing gas, the width of the EDL increases, leading to an increase in the sensor resistance. For *p-type* gas sensors, the width of the HAL decreases and increases upon exposure to reducing and oxidizing gases, respectively, leading to the appearance of a sensing signal. More details about the gas-sensing mechanism of resistive-based gas sensors will be discussed in the next sections. 

In brief, this review focuses on the different gas-sensing approaches used to enhance the sensing performance, which can provide the researchers with specific and targeted strategies for developing high-performance gas sensors. In this review, the state-of-the-art research on semiconductor-based chemiresistive-type gas sensors in Korea is described, with a focus on the research conducted by the laboratories of Professors Hyoun Woo Kim (Hanyang University) and Sang Sub Kim (Inha University). The success of our groups in the field of gas sensors stems from following items. (i) First, there is the synthesis of new materials with the desired morphology for sensing studies. For example, extensive efforts has been devoted to the synthesis of composite nanowires and nanofibers in core–shell (C-S) structure with different compositions. (ii) Second, there are advanced, almost automatic and precise measuring systems, where very small changes in the sensing signal can be detected and recorded by the system. (iii) Third, there is the optimization of sensing materials to obtain the highest sensing response. For example, the optimization of shell thickness or irradiation dose. (iv) Fourth, we focus on the finding the possible sensing mechanism by concrete evidence and using different characterization techniques. (v) Fifth, our success is the product of a highly motivated research group always monitoring the gas sensor-related literature, participating in gas sensor-related conferences, and collaborating with research groups all around the world. The studies performed in recent years on the core–shell, self-heated, irradiated, flexible, and Si/glass-based sensors are discussed. Accordingly, this review paper presents a lot of information about different gas sensors and their sensing mechanism.

## 2. Gas Sensors Based on Morphology Engineering: Core–Shell (C-S) Sensing Materials

Smart C-S nanocomposites, in which a core is covered with a nanoscale shell, have been subjected to sensing studies [19]. Different types of hybrid gas detection materials based on the C-S structure, such as MOS–MOS C-S, noble metal–MOS C-S, and noble metal–void oxide shell (yolk-shell), have been reported in the literature [64]. Among the MOS–MOS C-S type materials, one-dimensional (1D) C-S nanocomposites such as C-S nanowires (NWs) are well known for gas detection because these have large surfaces with a rapid diffusion pathway for gas molecules and provide a large number of heterojunctions, which are all important characteristics for realizing a good gas sensor [65]. Although there is a large difference in the structures of the nanofiber (NF) and NW sensors, a similar detection mechanism is likely involved in both cases. The results suggest that the radial modulation of the conduction channel is a common detection mechanism inside the C-S structures together with C-S NFs/NWs. In a C-S structure, when the shell thickness is equivalent to or less than the Debye length (λ_D_) of the shell layer, the whole of the shell is completely depleted from electrons due to (i) oxygen adsorption from air and (ii) flow of electrons at the C-S interface. Atomic layer deposition (ALD) is a suitable deposition method that allows the growth of smooth and high-quality films with excellent conformal on the underlying surface by controlling the thickness on the atomic scale at low temperatures [66,67]. Therefore, ALD is one of the best methods for depositing a layer of shell onto the core material. In addition, electrospinning is one of the simplest and most versatile techniques used for constructing a range of structures with different configurations, such as hollow, normal, porous, aligned, and C-S 1D nanostructures. This technique is very flexible for fabricating continuous and long NFs. It is expected that this will gradually advance from laboratory-scale to industrial-scale processes. From a marketing standpoint, electrospinning is the only preferred method for the large-scale fabrication of NFs compared to other existing techniques because of its ease of handling, low cost, minimum solution consumption, controllable NF diameter, and reproducible NF processing, in addition to technical advances compared to other techniques [68]. The following section is about the main results of the key research on the C-S nanostructure-based gas sensors published by the research groups led by Hyoun Woo Kim, Sang Sub Kim, and Yeon-Tae Yu (Jeonbuk National University, Jeonju, Korea). In most of this research, there is a detailed study about the mechanism of gas sensing in C-S-based gas sensors. 

**Figure 1:**
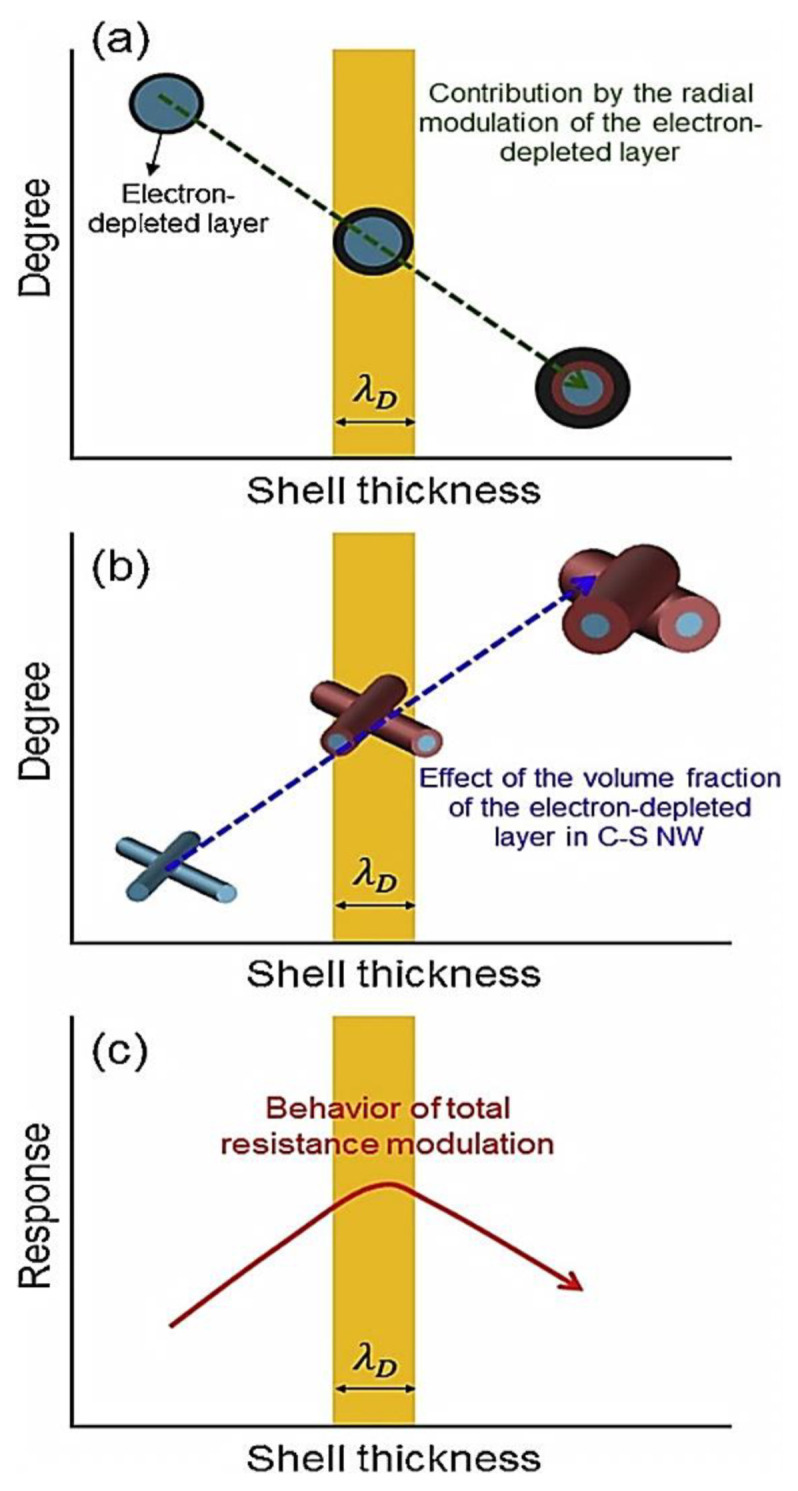
Effect of various mechanisms on the ZnO-SnO_2_ C–S NW sensor response: (**a**) radial modulation effect, (**b**) volume fraction effect, and (**c**) response variation as a function of shell thickness. Reproduced from [71] with the permission of Elsevier.

Park et al. [69] prepared TiO_2_-ZnO C-S NFs for oxygen sensing, wherein the shell thickness of ZnO grew almost linearly with the number of ALD cycles. Thus, the thickness of the shell layer could be readily controlled by adjusting the number of ALD cycles. The optimal sensor showed a response of ≈19 to 5000 ppm oxygen gas at 300 °C. Thus, the oxygen-sensing data suggested that the C-S NFs afforded a better response than the bulk- or film-type gas sensors because of the numerous surface adsorption sites for gaseous molecules owing to their higher surface-to-volume ratios. Choi et al. [70] fabricated SnO_2_-ZnO C-S NFs via a two-step approach, where SnO_2_ NFs were initially synthesized using an electrospinning technique, and ALD was subsequently used to deposit ZnO shell layers. The fabricated sensor exhibited a good response O_2_ gas at 300 °C. The response to 1000 ppm oxygen was ≈2, and it showed a detection limit of 70 ppm. The sensing enhancement relative to the pristine sensor could be attributed to a combination of homo- and heterointerfaces developed at the junctions within the C-S NF sensor. The increased response of the gas sensor was related to the resistance changes caused by the surface depletion layer of the C-S NF and potential barriers at the interfaces. Kim et al. [71] investigated the sensing properties of ZnO-SnO_2_ C-S NWs, where a SnO_2_ shell layer was deposited by ALD. Gas detection studies demonstrated that a sensor with an optimal shell thickness of 40 nm at 300 °C revealed good responses of 18.24, 14.94, and 16.46 to 1 ppm for CO, C_7_H_8_, and C_6_H_6_ gases, respectively. The degree of modulation of the electron-depleted shell was mostly dependent on the shell thickness (Figure 1a). When the shell thickness was almost equal to λ_D_, the electrons returned to the shell layer when they were exposed to the reducing gas, leading to the development of a partially electron-depleted shell from an original completely electron-depleted shell, resulting in a high response. In an air atmosphere, the shell was not completely depleted when the shell thickness was greater than λ_D_. Upon exposure to the reducing gas, the electron-depleted layer was reduced, and there was a slight change in the resistance, which resulted in a low gas response. Thus, in the case of C-S NWs, the shell thickness must be of the order of λ_D_ to achieve a high sensing response. In the case of a SnO_2_ shell with a thickness ≤λ_D_, the modulation of resistance should be nearly equivalent. However, the primary resistance values showed that the resistance increased with an increase in the shell thickness until the maximum value was obtained for a shell thickness of 40 nm.

Accordingly, along with radial modulation, other factors must be considered to determine the initial resistance and detection performance. For the C-S NWs, the portion of the total volume of the C-S NWs was proportional to the shell thickness (Figure 1b). Therefore, the overall combined reducing gas response could be envisioned as a bell-shaped curve, which was correlated to the shell thickness (Figure 1c). In reality, the net effect of the contributions from the two detection mechanisms mentioned above eventually determined the gas response of the C-S NW sensor, where the highest response was observed for the shell with a thickness of 40 nm. Together with the aforementioned mechanisms, the smearing effect related to the electric field must also be considered. For shell layers with thicknesses ≤ λ_D_, the electrical currents were not confined to the shell area and passed through the core and shell. In such a case, the shell was completely electron-depleted in ambient air and subjected to significant resistance modulation after exposure to the reducing gas. However, a significant proportion of the electron current also flowed through the NW core, resulting in a slight resistance change of the C-S NWs. However, for shell thicknesses ≥ λ_D_, the electrical currents most likely passed through the shell, and the resistance modulation was mainly governed by the radial modulation of the resistance. Choi et al. [72] synthesized C-S NWs via a two-step process, where core SnO_2_ NWs were primarily fabricated by a vapor–liquid–solid (VLS) growth process, and the ZnO shell layers were subsequently deposited using ALD. The SnO_2_-ZnO C-S NW sensor with a shell thickness of 40 nm showed an excellent response to different reducing gases such as benzene (*R_a_/R_g_* = 88 to 10 ppm C_6_H_6_), CO (*R_a_/R_g_* = 80 to 10 ppm CO), and toluene (*R_a_/R_g_* = 75 to 10 ppm C_7_H_8_) at 300 °C. However, the NO_2_-sensing responses of the sensors were decreased because of the formation of the shell layers.

**Figure 2:**
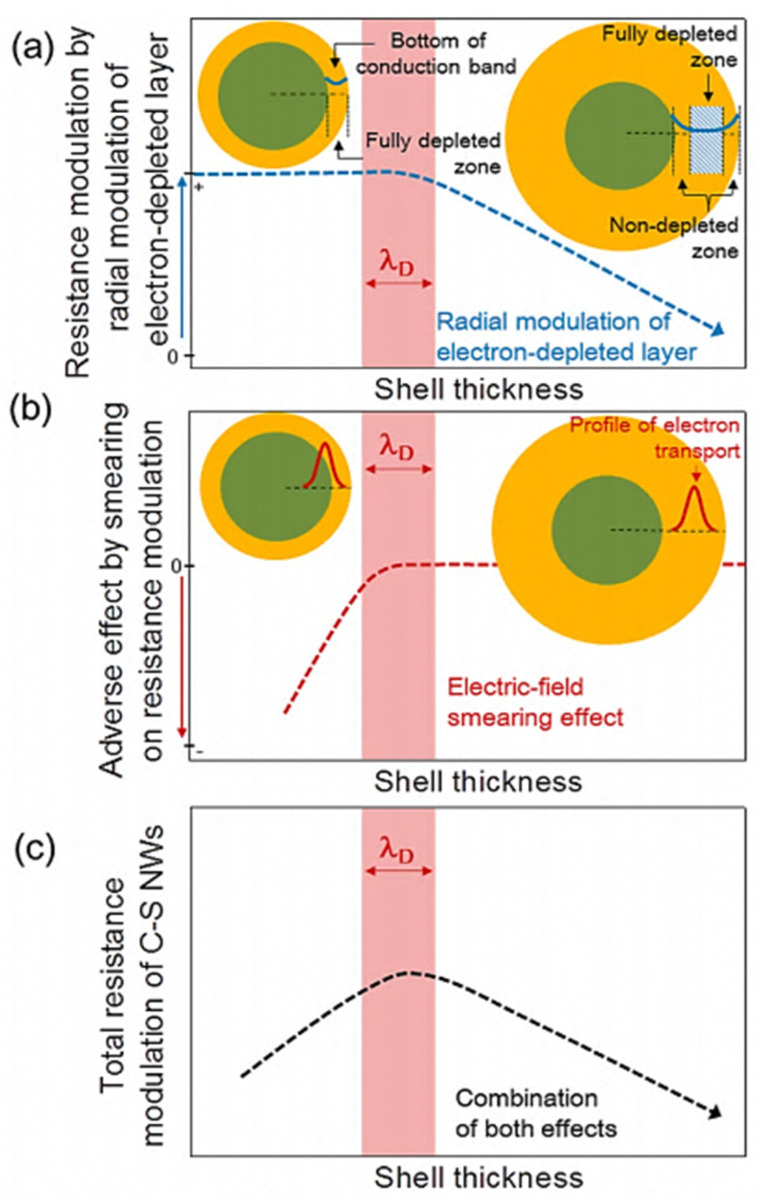
Conceptual explanation demonstrating the sensing mechanism of C-S NWs: (**a**) radial modulation, (**b**) adverse effect through smearing on resistance modulation, and (**c**) overall resistance modulation. Reproduced from [72] with the permission of the American Chemical Society.

The shells with thicknesses less than the Debye length are speculated to partially coat the core NWs, leading to a low gas response. Since the thickness of the shell is equal to or less than the Debye length of the shell layer, the modulation of resistance resulting from the release of electrons captured in the shell layer during the interaction of reducing gases must be nearly equal for all the C-S structures. However, for ZnO sensors with shell layers of thicknesses less than the Debye length, the modulation of resistance increases with increasing shell thickness up to the Debye length. This is in contrast to the interpretation based on the radial modulation mechanism of the electron-depleted shell layer. Accordingly, additional factors should be considered to account for the sensing outcomes. The degree of resistance modulation initiated by the radial modulation of the electron-depleted shell layer varies according to the shell thickness (Figure 2a). Figure 2b shows the sensing mechanism based on the electric field smearing effect. A thinner shell layer allows the development of the Debye length on the core material, and electrical transport is induced within the shell layer and around the C-S core interface because the passage is not restricted to the shell layer. This partially enhances the modulation of resistance when the C-S NWs are subjected to reducing gases because partial electrical transport is observed within the shell layer, which is an electrically depleted region. Even if the shell layer, which is completely depleted of electrons, undergoes a large modulation of the resistance, a considerable portion of electron transport is observed in the inner NW core, leading to a marginal modulation of resistance of the entire C-S NWs. In addition, for a shell layer thickness equal to or greater than the Debye length, the electrical transport is generally limited to the shell layer and not smeared to the core region. In such a case, the smearing effect becomes negligible, and the resistance modulation of the C-S NWs through reducing gases is governed by the radial modulation of the electron-depleted layer. The two above-mentioned effects cumulatively contribute to the gas response, as shown in Figure 2c.

**Figure 3:**
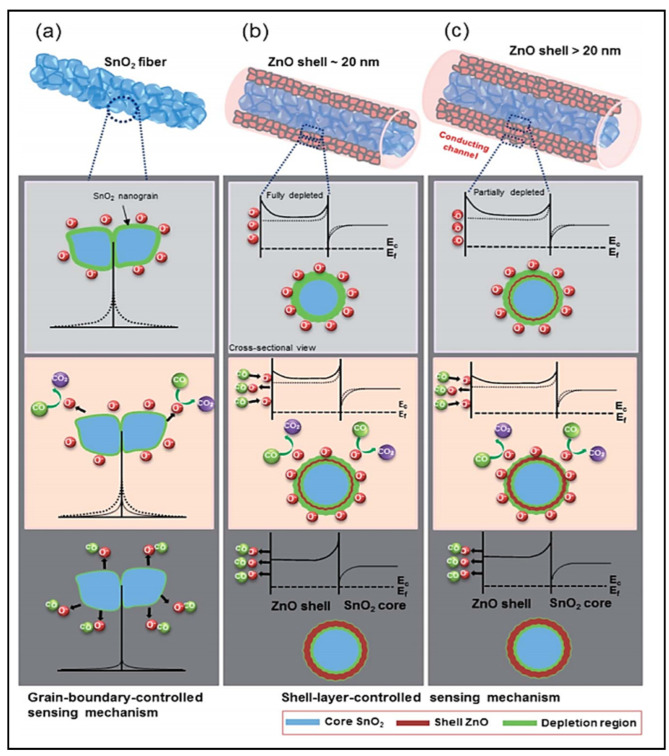
Schematic representation of the radial modulation of the ZnO shell layer: (**a**) pristine SnO_2_ NFs and SnO_2_-ZnO C-S with a shell thickness of (**b**) λ_D_ and (**c**) > λ_D_ [73]. Reproduced from [73] with the permission of Royal Society of Chemistry.

Katoch et al. [73] reported the synthesis of SnO_2_-ZnO C-S NFs via a combination of electrospinning and ALD techniques for CO and NO_2_ gas-sensing applications. The optimal gas sensor showed a response of 6.5 to 1 ppm CO gas at 300 °C, while the response to 1 ppm NO_2_ gas was 1.5. In the SnO_2_-ZnO C-S NFs, a heterojunction was formed between the core and shell, and the potential barrier of the heterojunction played an important role in enhancing the sensing capabilities of the C-S NFs. The height of the heterojunction potential barrier was modulated by reaction with the target gases. In the SnO_2_-ZnO C-S NFs, the shell layer was entirely electron-depleted when the thickness of the shell was equal to that of the shell material. The resistance of the pristine SnO_2_ NF sensor was significantly improved when a ZnO shell layer was created. In particular, the 20 nm-thick C-S NF sensor showed the maximum resistance, which indicated a completely depleted state of electrons in the ZnO shell. For the C-S NFs with shells of thicknesses greater than the Debye length, the resistance decreased again because of the partially depleted state of electrons. This change in resistance with varying shell thickness supported the electron transport mechanism in C-S, which was mainly controlled by the characteristics and thickness of the shell layer (Figure 3). The C-S NF sensors showed a low sensitivity to NO_2_ because of the unavailability of electrons in the shell layer. In the case of the C-S NFs with shell thicknesses greater than the λ_D_ of ZnO, the layer partially depleted by the electrons afforded a smaller number of electrons for the adsorbed NO_2_ gas molecules, which led to a larger resistance change than in the case of thick sensors [28]. 

**Figure 4:**
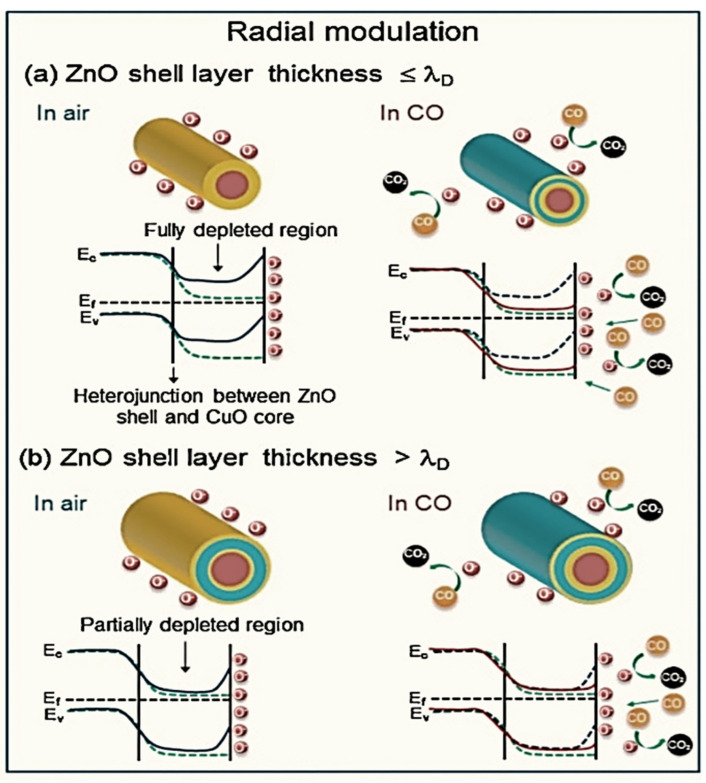
Schematic of the reducing gas-sensing mechanism of the CuO–ZnO C–S NW sensor for ZnO shell layers with thicknesses (**a**) less than *λ*_D_ and (**b**) greater than λ_D_. Reproduced from [74] with the permission of Elsevier.

Similarly, Kim et al. [74] investigated the gas-sensing characteristics of the *p-n* copper oxide (CuO)-ZnO C-S NWs toward the reducing gases, such as CO and C_6_H_6_ gases, where a sensor with a shell thickness equal to the λ_D_ of ZnO exhibited excellent sensing performance. The optimal gas sensor showed responses of 6 and 5.8 to 1 ppm CO and C_6_H_6_ gases, respectively at 300 °C with a detection limit of 1 ppm. For the shell layers with thicknesses equal to the λ_D_ of ZnO, the electrons from the ZnO shell were completely depleted owing to the combined effect of the *p*-*n* junction and oxygen adsorption on the shell. The sensing mechanism for the shell layer with a thickness less than λ_D_ is shown in Figure 4a. Notably, the shell thickness affording the highest sensing performance is almost equal to *λ*_D_, indicating a correlation between the sensing mechanism and *λ*_D_ value. When the sensors with a completely depleted shell layer were exposed to the reducing gases, the desorption surface oxygen released electrons into the conduction band of the shell to regain its original configuration, which significantly increased the conductivity of the C–S NWs. When the shell thickness was considerably smaller than *λ*_D_, the variation in resistance initiated mainly from the shell part was insignificant because of a small portion of the shell layer in the entire volume of the C–S NWs, even though the shell layer was fully electron-depleted and afforded a large variation in resistance. Thus, shells thinner than the critical value exhibited low a gas-sensing capability when compared to those with optimized thicker shells. In contrast, for the sensors with shell layers thicker than *λ*_D_, a slight change in resistance was observed (Figure 4b). Consequently, a shell with a thickness comparable to *λ*_D_ is crucial to achieve excellent performance for reducing gases.

**Figure 5:**
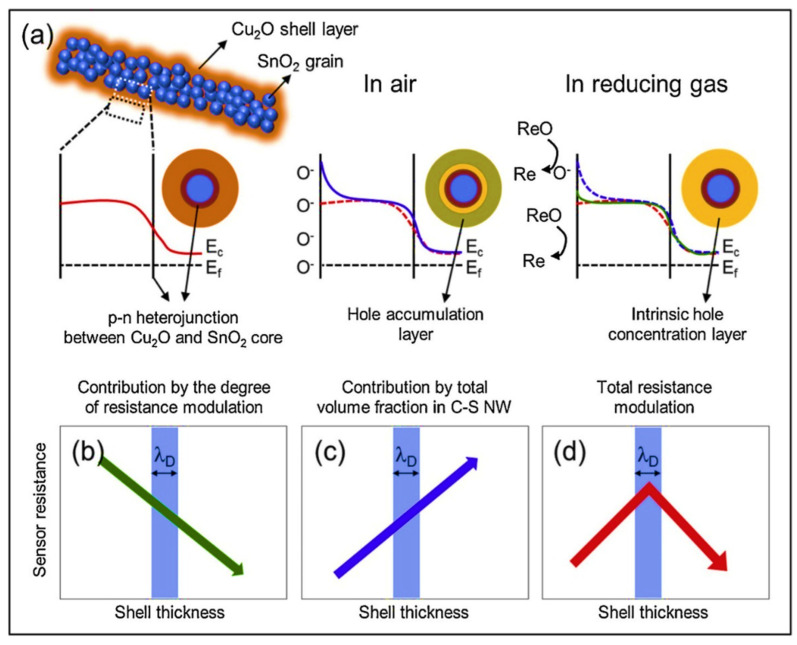
Schematic representation of the gas-sensing mechanisms of the SnO_2_-Cu_2_O C-S NF sensors. (**a**) Hole concentration profile and (**b**–**d**) variation in sensor resistance due to different contributions. Reproduced from [75] with the permission of Elsevier.

In another study, Kim et al. [75] synthesized SnO_2_/Cu_2_O C-S NFs by electrospinning followed by ALD of a Cu_2_O layer. The pristine SnO_2_ sensor showed the highest response toward NO_2_ gas (*R_g_/R_a_* = 5.3 to 10 ppm NO_2_ gas at 300 °C), and the C-S NF sensor with a shell thickness of 30 nm showed the maximum response to CO gas. The optimal gas sensor showed a response of 5.3 to 10 ppm CO gas at 300 °C. The sensing characteristics were not only affected by the initial concentration of holes in the shell but also by hindering the development of the hole-accumulation layers (HAL) (Figure 5a). The released electrons eradicated the holes and increased the shell layer resistance. In a shell of 15 nm thickness, the total layer was hole-accumulated, and hence, the subsequent transfer of holes from NO_2_ gas was not effective. Furthermore, the injection of CO gas eliminated the holes from the entire hole-accumulated layer and increased the resistance. For a shell thickness of 30 nm, a part of the shell was hole-accumulated by the adsorbed oxygen ions. The entire thickness was hole-accumulated by the adsorption of NO_2_ gas, which resulted in an enhanced sensor response. In addition, the accumulation of holes was decreased by the adsorption of CO gas, which increased the hole resistance, thereby affording a high sensor response. For an optimal shell thickness of 30 nm, it was assumed that the shell layer would be nearly hole-depleted upon CO incorporation, where the change in the hole resistance caused by the incorporation of CO gas would be maximized. Consequently, the sensor response increased for NO_2_ and CO gases with an increase in shell thickness beyond 15 nm. In contrast, the expansion of the rich-HAL layer was restricted by the presence of an *n-p* heterojunction, which acted as a blocking layer for expansion. Therefore, a further increase in the thickness of the shell resulted in low responses to NO_2_ and CO gases. For a shell thickness of 45 nm, the sensor response to CO gas was significantly decreased compared to that for a shell thickness of 30 nm. Additionally, the response to NO_2_ gas decreased slightly with a decrease in the shell thickness from 45 to 30 nm. For a shell thickness of 45 nm, the HAL layer was decreased to less than half the thickness of the shell layer. Therefore, a decrease in HAL through CO injection was insufficient, while an increase in HAL by injecting NO_2_ was sufficient. In addition, the sensing was not only affected by contribution from the degree of resistance modulation but also by the contribution of the overall volume fraction in the C-S NFs (Figure 5b–d). The resistance modulation caused by the radial modulation of the rich-HAL varied inversely with the shell thickness (Figure 5b). Along with this process, the degree of resistance modulation was higher in the thinner shells; however, the opposite was observed for thicker shells.

Regardless of other factors, it was expected that the thinnest shell would afford the highest gas response. However, sensing studies showed the dependency of the bell-shaped response on the shell thickness. An additional contribution was that of the fraction of the Cu_2_O shell to the overall volume of the C-S NFs, which was proportional to the thickness of the shell (Figure 5c). Therefore, the modulation of the total resistance showed a maximum at an exact thickness of the shell (Figure 5d). For a pristine SnO_2_ sensor, the NO_2_ response was greater than that of CO, whereas the developed C-S NF sensor showed a very poor response to NO_2_ in comparison to the pristine SnO_2_ sensors, indicating that the SnO_2_-Cu_2_O C-S NFs sensors were not effective in detecting NO_2_. The capability of NO_2_ gas for electron transfer from the Cu_2_O surface was significantly inferior to that of the SnO_2_ surface.

**Figure 6:**
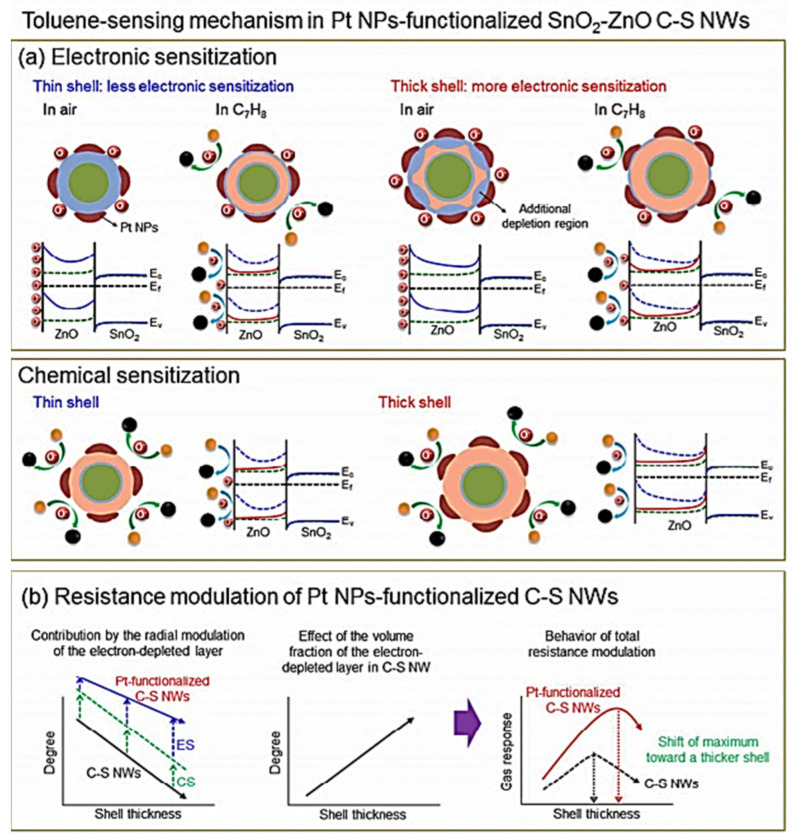
Schematic representation of the C_7_H_8_-sensing mechanism of the Pt-decorated SnO_2_-ZnO C-S NWs; (**a**) ES and CS effects of the Pt NPs and (**b**) shift of the response toward a thicker shell in the presence of Pt. Reproduced from [76] with the permission of the American Chemical Society.

Kim et al. [76] studied the effect of Pt functionalization with an the optimal shell thicknesses on the C_7_H_8_-sensing characteristics of SnO_2_-ZnO C-S NWs. The sensor showed a high response of 279 to 0.1 ppm C_7_H_8_ gas at 300 °C. Functionalization using Pt NPs resulted in two contributions to the sensing characteristics of the SnO_2_-ZnO C-S NWs. The first contribution was related to the electronic sensitization (ES) that was initiated by the electron flow from the ZnO shell layer to the Pt NPs, which led to a further suppression of the conduction channels configured inside the layer of the ZnO shell (Figure 6a). 

When the C_7_H_8_ molecules reacted with chemisorbed oxygen species, the width of the depletion layer changed, resulting in the resistance modulation of the gas sensor. The second contribution was the catalytic effect of the Pt NPs through chemical sensitization (CS), which allowed further interactions between the C_7_H_8_ molecules and chemisorbed oxygen species. The ES and CS effects are shown in Figure 6a. The ES contribution depended only on Pt NPs, and accordingly, the CS contribution was mainly accountable for selective sensing because of various phases of catalytic interactions between the metal NPs and a specific gas. As shown in Figure 6b, owing to the ES and CS contributions of the Pt NPs, the gas response of the sensor based on Pt-functionalized SnO_2_-ZnO C-S NWs was further enhanced, and the response maxima shifted toward a thicker shell. As shown in Figure 6, the gas response of the Pt-functionalized SnO_2_-ZnO C-S NWs increases linearly with an increase in the shell thickness, unlike the SnO_2_-ZnO C-S NWs without Pt NPs. The Pt NPs could dissociate the C_7_H_8_ gas molecules more efficiently than the other gases. The adsorption of C_7_H_8_ gas molecules onto the Pt surface was largely affected by the electronic effects, which decreased the barrier for C_7_H_8_ adsorption by making it easier to donate the electrons from the πCH_3_ level to the Fermi level and readily allowing back-donation from the Fermi level to the πCH_3_* level.

**Figure 7:**
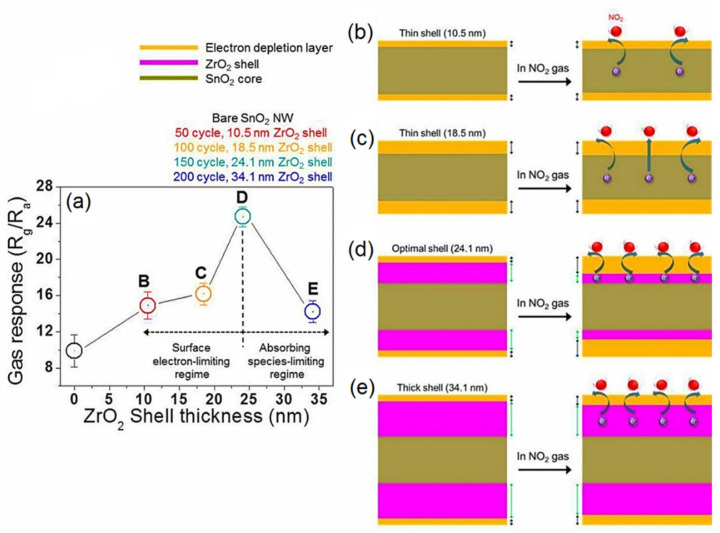
(**a**) NO_2_ responses of SnO_2_-ZrO_2_ C-S NW sensors as a function of ZrO_2_ shell thickness. (**b**–**e**) Illustration of NO_2_ sensing by the gas sensors with ZrO_2_ shell thicknesses of (**b**) 10.5, (**c**) 18.5, (**d**) 24.1, and (**e**) 34.1 nm. Reproduced from [78] with the permission of Elsevier.

Zirconium oxide (ZrO_2_) is typically utilized for electrochemical gas sensing at high temperatures [77], and the number of reports on its resistive-based gas sensing is limited. Bang et al. [78] synthesized–SnO_2_-ZrO_2_ C-S NWs with different shell thicknesses (10.5–34.1 nm) and studied their NO_2_ gas-sensing properties (Figure 7a). 

The optimal sensor showed a response of 24.7 to 10 ppm NO_2_ gas at 150 °C. The schematics showing NO_2_ gas sensing with various shell thicknesses are shown in Figure 7b–e. With an increase in the shell thickness of ZrO_2_, the number of electrons in ZrO_2_ increased in the range of 0–24.1 nm. As the r esponse of ZrO_2_ increased with respect to the electron conduction volume, the developed sensor system in the range of 0–24.1 nm was in command, where the surface electron concentration of ZrO_2_ was lower than that of the adsorbing gas species. In this surface-electron-limiting regime, most of the available surface electrons interact with the adsorbed gaseous species. Accordingly, an increase in the volume of electron conduction led to an increase in the sensor response. For a shell thickness less than the optimum value, almost the entire thickness was depleted. Upon NO_2_ exposure, insufficient electrons were extracted from the ZrO_2_ layer. Accordingly, the number of surface electrons was inadequate in the sensor with thin shells, and consequently, the response of the sensor was restricted. Consequently, the detection capability improved with an increase in the shell thickness from 0 to 24.1 nm. At an optimum shell thickness of 24.1 nm, the shell layer of ZrO_2_ was significantly depleted by electrons. Upon exposure to NO_2_ gas, the remaining electrons from ZrO_2_ were completely depleted by the adsorption of NO_2_ species. The initial concentration of electrons was extremely high for the thin shells; therefore, a large number of electrons were consumed during the reactions with NO_2_ gas. The sensor response decreased with an increase in the shell thickness from 24.1 to 34.1 nm because of the presence of adequate surface electrons above 24.1 nm owing to the thick shell of ZrO_2_. This modified the system to afford a regimen in which the concentration of surface electrons in ZrO_2_ was greater than that of the adsorbed gaseous species. In such an adsorbing-species-limiting regimen with a high initial conduction volume, a decrease in the ZrO_2_ surface electrons upon the injection of the target gas resulted in a low sensor response, which was achieved through a thick shell layer. Further increase in the shell thickness resulted in a lower sensor response. Relative to the optimized shell thickness, a large portion of the shell layer was not depleted by the electrons. Even though NO_2_ adsorption led to the adsorption of some electrons, the relative change in the electron concentration within ZrO_2_ was not very significant, resulting in a relatively lower sensor response.

**Figure 8:**
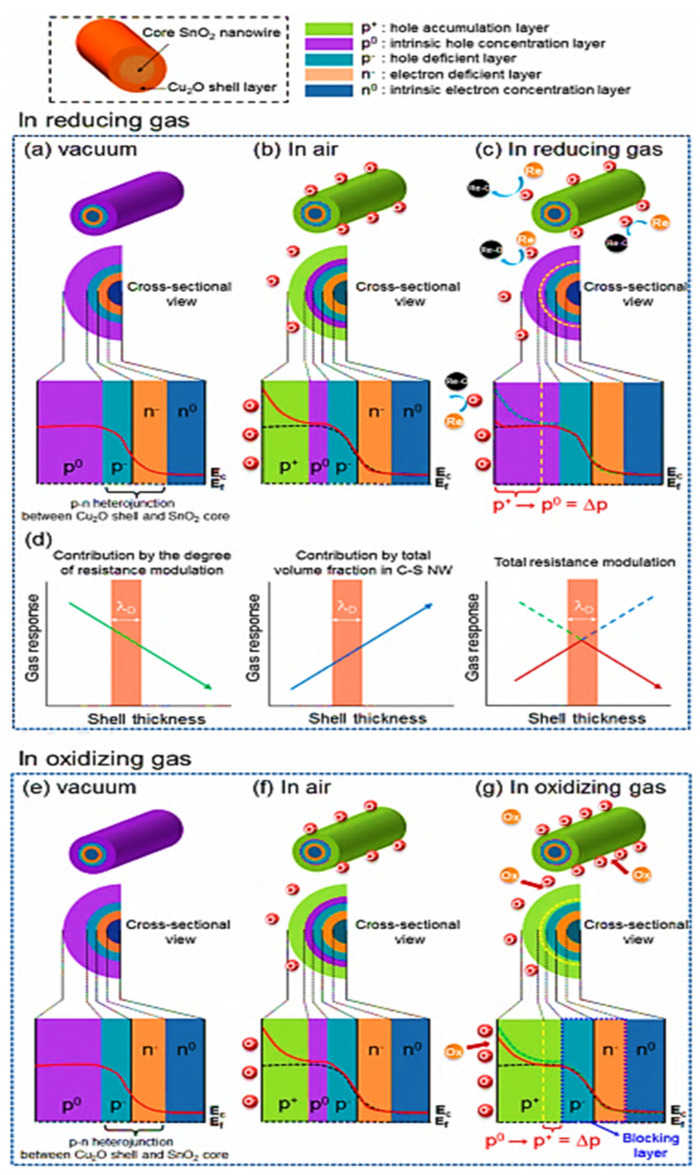
Conceptual explanation of the sensing mechanism in the SnO_2_−Cu_2_O C-S NWs; (**a**) in vacuum, (**b**) in air, and (**c**) in the presence of reducing gases, and (**d**) overall modulation of the resistance. The sensing mechanism for oxidizing gases in (**e**) vacuum, (**f**) air, and (**g**) oxidizing gases. Reproduced from [79] with the permission of the American Chemical Society.

Kim et al. [79] synthesized SnO_2_-Cu_2_O C-S NWs and applied these to the detection of trace amounts of gases. The resistance curves for the C-S NW sensors with different shell thicknesses were obtained upon exposure to 10 ppm C_7_H_8_, C_6_H_6_, and NO_2_. The sensor based on C-S NWs with a shell thickness of 30 nm exhibited the best response to reducing gases. The response of optimal gas sensor to 10 ppm C_7_H_8_, C_6_H_6_ gases was 11.7, 12.5 at 300 °C. In addition, the response and recovery times were almost 4 s for both gases. The presence of the Cu_2_O shell decreased the NO_2_-sensing response of the C-S NW sensors. The sensing mechanism of the SnO_2_-Cu_2_O C-S NW sensor is shown in Figure 8. In ambient air, the concentration of holes can be divided into three regions (considering the vacuum case (Figure 8a) because of oxygen adsorption onto the Cu_2_O shell and development of the C-S heterojunctions (shown in Figure 8b). The HAL (*p*^+^) is created by the extraction of electrons from the valence band of Cu_2_O by chemisorbed oxygen species. At a specific temperature, the intrinsic hole concentration layer (*p_o_*) remains at the equilibrium hole concentration in Cu_2_O, and the hole-deficient layer (*p*^−^) results from an electrostatic response to the hole layer by the electrons in the *n-p* heterojunction. The red line in Figure 8b shows the profile of the hole concentration in ambient air, and the dotted black line shows the case in vacuum. An increase in the concentration of holes is observed in air. When the sensor is exposed to the reducing gas, the resistance of the *p*-Cu_2_O shell layer increases. As shown in Figure 8c, the profile of hole concentration (blue line) in air shifts toward the red line, which supports a decrease in the concentration of holes in the “*p*” shell layer. Therefore, the detection capability of pure Cu_2_O NWs was inferior to that of the C-S NWs because of the weaker hole-accumulation layer. The degree to which the resistance of the *p*^+^ layer is modulated varies inversely with the shell thickness. As a result, a thicker shell experiences less resistance modulation because it is in a state of partial hole accumulation. Considering the fraction of shell layers in the overall volume of the *n-p* C-S NWs (which is comparable to shell thickness), the response affords a bell-shaped curve as a function of shell thickness (Figure 8d). As shown in Figure 8e–g, the extension of the *p*^+^ layer is constrained owing to the existence of the *p*^−^|*n*^−^ interface, which acts as a blocking layer for the expansion of the *p*^+^ layer, resulting in a slight resistance modulation to oxidizing NO_2_ and low response to NO_2_.

**Figure 9:**
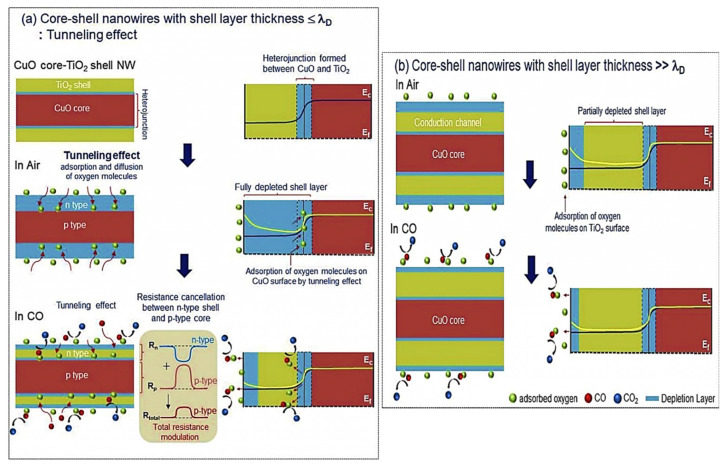
Schematic of the detection mechanism of CuO-TiO_2_ C-S NWs with the shell layer thickness of (**a**) < λ_D_ and (**b**) > λ_D_ [80]. Reproduced from [34] with the permission of Elsevier.

In another study, Lee et al. [80] reported the gas-sensing characteristics of *p-n* CuO-TiO_2_ C-S NWs, where a sensor with a 40 nm shell thickness exhibited an enhanced gas response (*R_a_/R_g_* = 7.14 to 1 ppm CO at 300 °C). The TiO_2_ shell layer was depleted by electrons through oxygen adsorption together with the transfer of electrons to CuO (Figure 9a). The degree of electron depletion increased when the shell thickness was below the λ_D_ of CuO. Specifically, a depleted state of electrons was formed in the thin-shell layer. In contrast, only partial electron depletion was observed for thick shells (Figure 9b). Therefore, the degree of resistance modulation initiated by radial modulation within the shell layer varied inversely with the shell thickness. The thinner shells underwent further resistance modulation, whereas the thicker shells showed less resistance modulation with the partial depletion of electrons in the shell. When the TiO_2_ shell thickness was greater than 40 nm, the shell prevented the gas molecules from interrelating with the core CuO as it was sufficiently thick to act as a barrier layer. In this state, the sensor based on C-S NWs exhibited *n-*type sensing characteristics because the TiO_2_ shell layer was the only sensing material subjected to gas molecules. 

Katoch et al. [81] synthesized *p*-CuO/*n*-ZnO C-S NFs for CO gas-sensing applications. In the p-n CuO/ZnO C-S NFs, the shell layer was completely depleted by the electrons when the shell thickness was equivalent to the Debye length of the shell material. This confirmed that the C-S NF sensors showed a better response when the shell thickness was lower than that of the Debye length of the shell material. The highest sensor response to CO was observed with a 16 nm-thick shell (Response = 7 to 0.1 CO at 300 °C), suggesting that the *p-n* C-S NFs with 16 nm-thick shells were completely depleted. When a fully depleted *p-n* C-S NF shell layer was exposed to CO, the oxygen species that were eliminated from the surface released electrons into the conduction band of the shell layer and the original configuration of the band was recovered, thereby increasing the conductivity. In addition, the *p-n* C-S NFs with a shell layer thickness greater than the Debye length only had partially depleted shell layers. Upon exposure to CO gas, the CO molecules were adsorbed onto the partially electron-depleted shell layer. A slight change in resistance was observed because of the presence of a conduction channel below the partially electron-depleted shell layer. This indicated that the shell thickness modulated the electron-depleted layer in the shell. Therefore, preparing a shell with a thickness less than or equivalent to the Debye length is important to achieve excellent gas-sensing properties of the *p-n* C-S NF sensors. Notably, the *p-n* C-S NF sensors did not have a better capability to detect oxidizing gases than normal NFs. Generally, oxidizing gases extract many electrons from the shell surface. Considering that *n-*type shells contain free electrons throughout their partial volume irrespective of the type of core nanofiber, these are simply depleted. Therefore, the free electrons are depleted just before or immediately after introducing the oxidizing gas, leading to a change in the resistance and a low response. Thus, the lower response to oxidizing gas is mainly due to the inadequate accessibility of free electrons in the completely depleted shell layer for inbound oxidizing gas molecules. Similarly, Park et al. [82] introduced a model to improve the gas-sensing capacity of SnO_2_-ZnO C-S NFs. The ZnO shell was completely depleted by the electrons via the combined effect of (i) band bending at the heterojunction between the ZnO shell layer and the core SnO_2_ fiber and (ii) the band bending at the ZnO shell layer surface through the adsorption of oxygen molecules. Upon CO exposure, the released electrons gradually recovered to the original configuration of the band. Through this process, the resistance change along the NFs was more noticeable than the normal NFs without a fully depleted layer owing to the absence of the completely depleted shell layer. 

**Figure 10:**
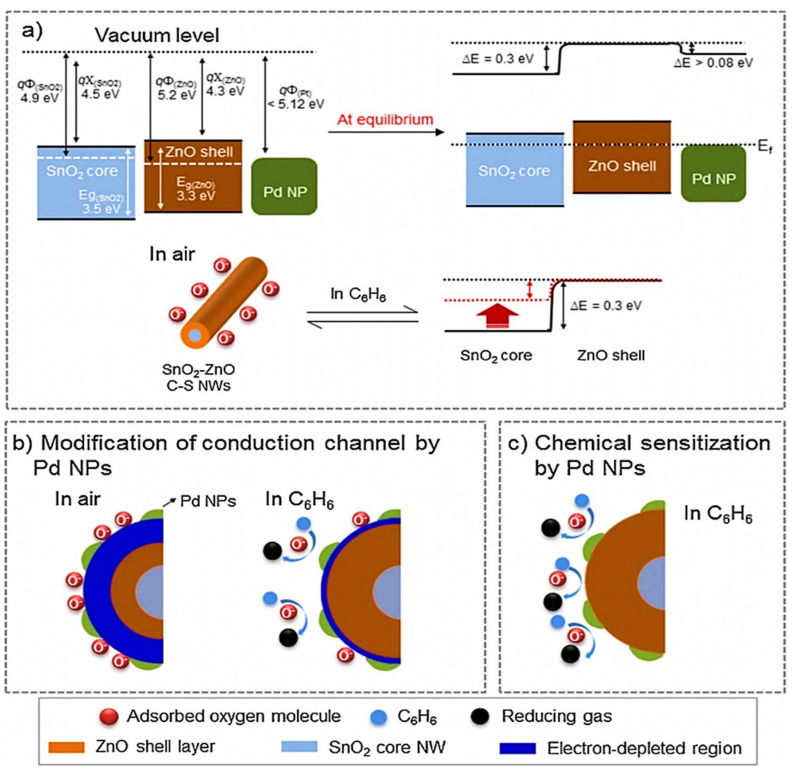
(**a**) Schematics of the energy levels of ZnO, SnO_2_, and Pd, (**b**) conduction channel modification in the shell layer, and (**c**) the CS effect achieved through Pd NPs. Reproduced from [83] with the permission of Elsevier.

In another study, Kim et al. [83] achieved outstanding selective sensing of C_6_H_6_ gas using Pd NP-functionalized SnO_2_-ZnO C-S NWs. The authors reported an excellent response of 71–100 ppb C_6_H_6_. The C-S NWs showed enhanced sensing properties toward reducing gases for the optimized shell thickness with respect to the pristine core NWs. A potential barrier was formed at the heterointerfaces (Figure 10a) because of the different work functions of different materials via charge transfer. The sensor response was attributed to the changes in the potential barriers in air and C_6_H_6_ gas atmosphere. An additional contribution to the detection signal was the modification of the conduction channel by Pd NPs (Figure 10b). Furthermore, specific attention should be paid to CS through the catalytic effect of the Pd NPs (Figure 10c), which facilitate C_6_H_6_ interactions with the chemisorbed oxygen species and enhance the resistance modulation of the SnO_2_-ZnO C-S NWs. The Pd NPs are likely to more efficiently dissociate C_6_H_6_ gas molecules than other reducing gases. The catalytic properties of Pd allow the adsorption of C_6_H_6_ molecules to Pd NPs.

**Figure 11:**
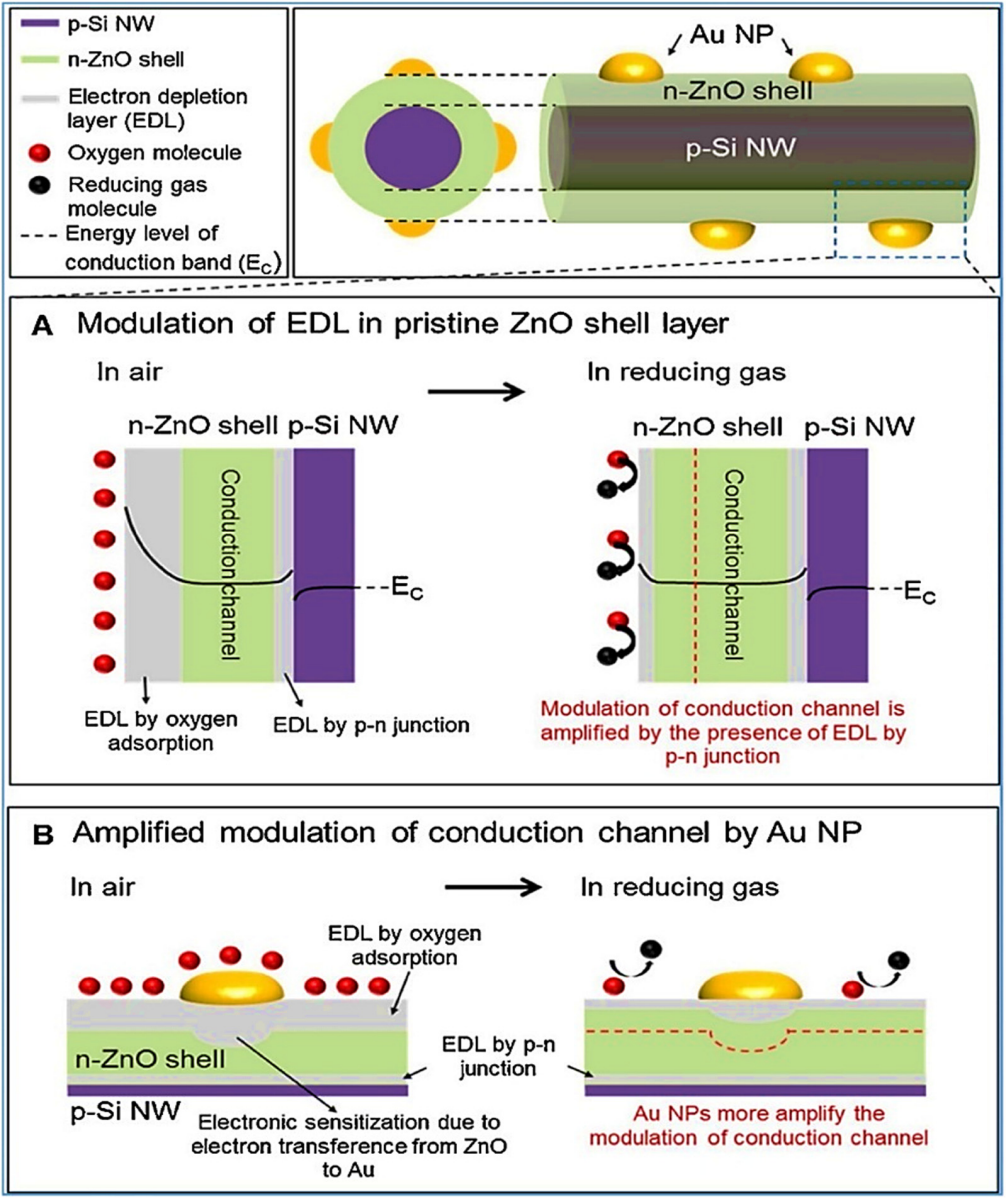
Schematic of the sensing mechanism in the Au-decorated Si NWs/ZnO C-S sensor; (**A**) depletion layers in pure Si NWs/ZnO C-S layer, and (**B**) expansion of electron depletion layer in the presence of Au. Reproduced from [84] with the permission of Elsevier.

In addition, Choi et al. [84] utilized Au-decorated Si NW-ZnO C-S for H_2_S gas-sensing applications. The optimal gas sensor showed a response of 11 to 50 ppm H_2_S at 300 °C. The response and recovery times were 48 and 63 s, respectively. A possible sensing mechanism for the Au-decorated Si NWs-ZnO C-S is shown in Figure 11. Various factors were considered for the elucidation of the sensing mechanism, including (i) ZnO/Au heterojunctions, (ii) catalytic properties of Au, and (iii) the heterojunction between ZnO/Si. As a result of the catalytic activity of Au, gas molecules dissociated, and the spillover effect was transferred onto the ZnO shell surface. Furthermore, the increase in the sensor response by increased electron flow in ZnO-Si-ZnO occurred when the ZnO work function was higher than that of *p*-Si. In addition, the ZnO/Au heterojunction barriers increased the sensor performance. The sensor displayed better selectivity toward H_2_S gas because of its high reactivity, and therefore, it could simply react with the chemisorbed oxygen ions on the ZnO surface. In addition, the bond energy between H and SH in H_2_S is 381 KJ/mol, which is lower than those of other interfering gases. As a result, the H-SH bond may readily collapse at 300  °C during chemical adsorption. Although the bond energy of O-NO is less than that of H-SH, the interaction strength between the sensor and target gas is another significant parameter that allows the determination of the gas response. The adsorption of H_2_S gas molecules onto Au NPs may potentially result in the formation of Au-S or Au-SH-type species on the Au NP surface. Consequently, a sulfide shell covered the surfaces of the Au NPs, which decreased the surface work function of the Au NPs. According to this change, the amount of electron exchange between the Au NPs and ZnO could be changed, which could improve the sensor response to H_2_S gas.

**Figure 12:**
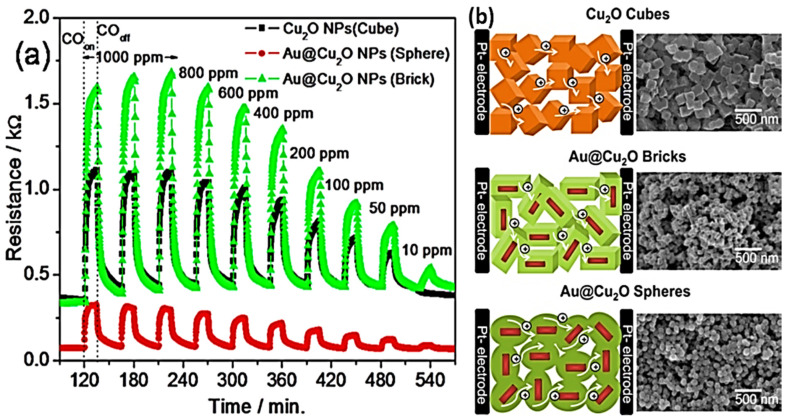
(**a**) Dynamic resistance curves of different Cu_2_O gas sensors for CO gas and (**b**) the sensing mechanism of different gas sensors. Reproduced from [89] with the permission of Elsevier.

Another type of C-S structure relies on noble metals and metal oxides. Pioneering research on noble metal–metal-oxide C-S based gas sensors was first developed by Prof. Yeon-Tae Yu et al. at Jeonbuk National University, Korea. This research group successfully developed a series of noble metal and metal–oxide-based C-S gas-sensing materials, such as Au-SnO_2_ [85,86,87], Au-Cu_2_O [88,89], Au-ZnO [90], Au-NiO [91], PdO-ZnO [92], Au-In_2_O_3_ [93], and AuPd_alloy_-ZnO [94]. Among various C-S sensors, the first type of C-S gas sensor based on Au@SnO_2_ C-S nanoparticles (NPs) was reported by Yu et al. in 2011 [85]. The authors synthesized Au-SnO_2_ C-S NPs via a microwave-assisted hydrothermal reaction and investigated their CO gas-sensing properties. The response (*R_a_/R_g_*) of the Au-SnO_2_ C-S NPs was ≈1 for 1000 ppm CO at 100 °C. It was speculated that the catalytic activity of Au NPs was responsible for the superior response of the Au/SnO_2_ C-S NPs at temperatures below 200 °C compared to pure SnO_2_ NPs [95]. To further enhance the sensing properties of C-S NPs, Majhi et al. [89], synthesized three kinds of Cu_2_O NPs in 2014, namely cubic Cu_2_O NPs, spherical Au NRs-Cu_2_O C-S, and brick-shaped Au NRs-Cu_2_O C-S NPs for CO gas-sensing applications. Figure 12a shows the CO gas-sensing performances of the three types of Cu_2_O NPs at 250 °C and concentrations of 10–1000 ppm. The brick-shaped Au-Cu_2_O C-S NP sensor exhibited a typical *p*-type semiconductor behavior with the highest response of 5.67 (*R_g_/R_a_*), which was followed by 4.38 for the spherical-shaped Au-Cu_2_O C-S and 3.35 for pristine cubic Cu_2_O NPs. As shown in Figure 12a, significant variations in baseline resistance are observed, which are attributed to the unique structure of the Au-Cu_2_O C-S NPs, ES of the Au NRs, and arrangement of particles between the two electrodes. Figure 12b shows a schematic of the flow of electrons in the three types of sensing layers with their corresponding microscopic images. Spherical Au NRs-Cu_2_O C-S NPs were well connected to each other because of their spherical geometry, which facilitates the charge flow and results in a low baseline resistance.

**Figure 13:**
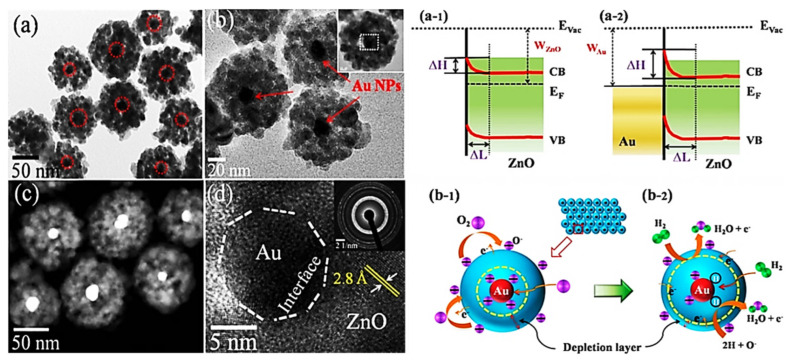
(**a**–**d**). TEM images of Au-ZnO C-S NPs and ((**a-1**)–(**b-2**)) the corresponding H_2_ gas-sensing mechanism of Au-ZnO C-S NPs. Reproduced from [90] with the permission of Elsevier.

The same authors further examined the H_2_ gas-sensing properties of Au-ZnO C-S NPs [90]. Au-ZnO C-S NPs were synthesized using a low-temperature hydrothermal method (Figure 13a–d). The NP size of Au-ZnO C-S was 50–70 nm, together with a size 10–15 nm of the Au NPs encapsulated inside the materials. The sensors exhibited the highest response (*R_a_/R_g_* = 103.9) in comparison to pristine ZnO NPs (12.7) with a rapid response time of 75 s and a low detection limit of 500 ppb. The sensor also showed good reproducibility and stability toward H_2_ gas when tested in repeated cycles. The enhanced sensing properties were attributed to the electronic and CS effects of the Au NPs (Figure 13((a-1)–(b-2))) [96].

**Figure 14:**
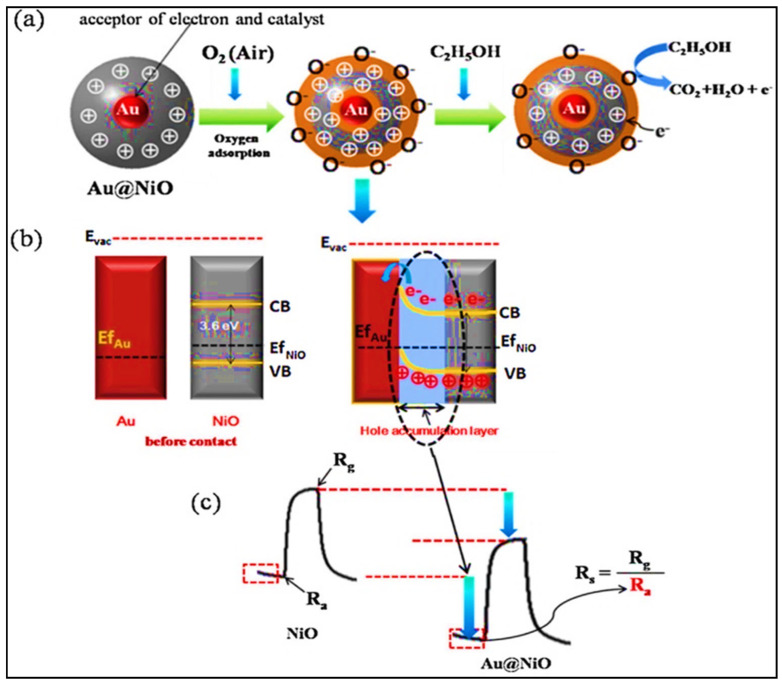
(**a**) C2H5OH-sensing mechanism of Au-NiO C-S NPs. (**b**) Formation of heterojunctions between Au and Ni (**c**) comparison of sensor signal in NiO and Au-NiO C-S NP gas sensors. Reproduced from [91] with the permission of Elsevier.

Majhi et al. [91] further synthesized *p*-type Au-NiO-based C-S NPs and investigated their ethanol (C_2_H_5_OH)-sensing properties at low temperatures. The highest response of Au-NiO C-S NPs toward 100 ppm C_2_H_5_OH was 2.54 in comparison to 1.88 for pristine NiO NPs. The operating temperature of Au-NiO C-S NPs was lower (200 °C) than that of the pristine NiO NPs (300 °C). The formation of Au-NiO heterojunctions was responsible for the enhanced response to C_2_H_5_OH gas (Figure 14c). Majhi et al. [92] also reported a *p-n* heterojunction based on PdO-ZnO C-S NPs for acetaldehyde gas sensing. The as-prepared PdO-ZnO *p-n* heterojunction C-S NPs were tested for many gases, and the highest sensing response of 76 (*R_a_/R_g_*) was observed for 100 ppm acetaldehyde gas at 350 °C in comparison to that for pristine ZnO NPs (*R_a_/R_g_* = 18). The enhanced sensitivity of PdO-ZnO C-S NPs was attributed to the formation of a *p-n* heterojunction structure, the catalytic effect of PdO NPs, and the high surface area of the obtained materials. Recently, Yu et al. investigated the sensing properties of noble metal alloy-based metal–oxide C-S NPs [94]. The authors synthesized AuPd_alloy_@ZnO C-S NPs and examined their H_2_ gas-sensing properties. Different compositions of AuPd alloys, such as Pd_20_Au_80_, Pd_35_Au_65_, and Pd_50_Au_50_, were individually used to prepare C-S NPs. When tested for different gases, the Pd_35_Au_65_-ZnO C-S NP-based sensor showed an enhanced response toward 100 ppm H_2_, among other gases, with a high selectivity at 300 °C. This was attributed to the synergistic effect of the AuPd alloy and ZnO with the ES effect and the formation of the PdH_x_ component due to the reaction with dissociated hydrogen atoms [94].

Another research group from Korea University studied the gas-sensing applications of different C-S and yolk–shell NPs [97,98]. For example, Rai et al. reported a yolk–shell-type structure based on Pd@In_2_O_3_ and studied its C_2_H_5_OH gas-sensing properties. The highest response of 159.02 toward 5 ppm C_2_H_5_OH was observed at 350 °C, which was 10 times higher than that of pristine In_2_O_3_ NPs. Similarly, the same group reported the synthesis of an additional type of *p*-type Au-NiO yolk–shell NPs and studied their gas-sensing properties toward H_2_S gas [99]. The response of Au-NiO yolk–shell NPs to 5 ppm H_2_S was 108.92, which was almost four times higher than that of pristine NiO hollow nanospheres. The improved performance was attributed to the unique hollow structures of the Au-NiO yolk–shell NPs, which facilitated the accessibility of Au NPs. The H_2_S adsorption on Au NPs resulted in the formation of a sulfide layer, which likely decreased the work function of the Au-NiO yolk–shell NPs. As a result, electron transfer occurred from Au to NiO rather than NiO to Au, which increased the resistance of the sensor and response to H_2_S. Rai et al. [97] further investigated C-S NPs based on Ag-SnO_2_ NPs. The Ag-SnO_2_ C-S NPs were synthesized using a microwave-assisted hydrothermal method. The as-prepared sensor exhibited a high response of 16.17 (*R_a_/R_g_*) to 5 ppm *p*-xylene among other interfering gases, whereas the response of pristine SnO_2_ NPs was only 10.79. The enhanced *p*-xylene-sensing performance of Ag-SnO_2_ C-S NPs was correlated to the electronic and CS effects of the Ag NPs. Therefore, it is concluded that the functionalization or encapsulation of noble metal NPs has a significant role in enhancing the sensing performance.

## 3. Self-Heated Gas Sensors

The major feature of self-heating gas sensors is the application of an appropriate voltage. The Joule heating effect is mainly caused by small-scale heat dissipation. Any obstacle that leads to electron deviation from its pathway may result in the production of heat. Heat is generated within the sensor owing to the Joule heating process, which results in an increase in its temperature [100]. In the case of Joule heating, heat is generated owing to the kinetic energy loss of the electrons through collisions between the lattice atoms and moving electrons [101]. Operation at room temperature is one of the major advantages of self-heated gas sensors. In the following section, the results of the most relevant papers in the field of self-heated gas sensors published by the laboratories of Professors Hyoun Woo Kim and Sang Sub Kim are discussed.

**Figure 15:**
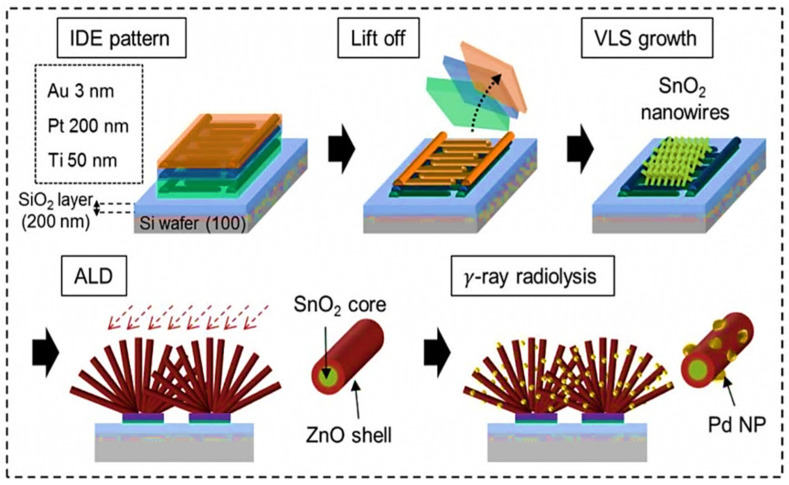
Schematic of the synthesis of Pd-decorated SnO_2_-ZnO C-S NWs. Reproduced from [104] with the permission of Elsevier.

Pd is mostly utilized as a C_6_H_6_-sensing agent owing to its good catalytic activity [102]. In addition, NW-type one-dimensional morphologies are highly favored for self-heating studies [103]. Kim et al. [104] utilized Pd-functionalized SnO_2_-ZnO C-S NWs (C-S NWs) for sensing C_6_H_6_ gas in the self-heating mode. A high response of 71 at 100 ppb of C_6_H_6_ gas was attained under 20V applied voltage. Figure 15 shows a schematic of a three-step synthesis method comprising the growth of SnO_2_ NWs via the VLS mechanism, subsequent ZnO deposition by the ALD technique, followed by gamma ray irradiation for Pd deposition. 

**Figure 16:**
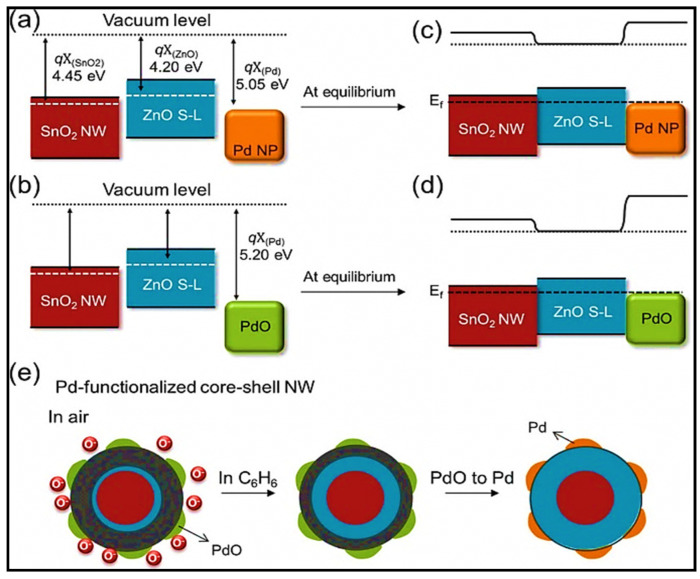
(**a**–**d**) Schematic of the energy levels of SnO_2_-ZnO-Pd and SnO_2_-ZnO-PdO systems and the development of heterojunction barriers. (**e**) Change in depletion layer width in the presence of Pd. Reproduced from [104] with the permission of Elsevier.

The as-fabricated sensors exhibited an improved C_6_H_6_ response in the self-heating mode. An enhanced response of 71–100 ppb of C_6_H_6_ gas was obtained at an applied voltage of 20 V. The three Joule heating sources in the sensors included the (i) electron collisions with one another and with Zn and O atoms in the ZnO grain, (ii) the collision of electrons and ZnO grain boundaries, and (iii) kinetic energy loss of electrons in ZnO–ZnO homojunctions, whose structure was dense and tangled. As a result of the different work functions of ZnO, SnO_2_, Pd, and PdO, potential barriers were developed at the interfaces between these materials (Figure 16a–d), which acted as a source of the resistance within the gas sensor upon exposure to C_6_H_6_ gas. Furthermore, Pd NPs exhibited promotional effects on the gas detection properties of C_6_H_6_ gas. In CS, Pd initiated the dissociation of molecular oxygen in the atomic form. Accordingly, Pd increased the reaction rate of the C_6_H_6_ gas molecules. Through the spillover effect, C_6_H_6_ molecules were primarily adsorbed onto the Pd NP surface and then transferred to the ZnO surface to respond with the chemisorbed species. Therefore, further reactions resulted in a higher C_6_H_6_ response.

Tungsten disulfide (WS_2_) is one of the most important members of the *2D* transition metal dichalcogenides (TMDs) and has unique properties such as low cost, good thermal stability, and tunable band structure. In addition, the weak van der Waals forces attach the vertically stacked layers in WS_2_, which simplifies the diffusion of target gases between the layers [105]. Nonetheless, gas sensors based on pure WS_2_ exhibit poor selectivity, low sensitivity, and long recovery time, which limits their applications for realistic purposes [106]. Kim et al. [107] described the CO gas-sensing features of Au-functionalized *2D* WS_2_ nanoflakes in the self-heating mode (1–5 V), where Au was deposited over WS_2_ using UV light for different durations of irradiation. One of the objectives was to investigate the amount of Au on the WS_2_ surface for CO gas detection in the self-heating mode. The developed sensor exhibited a greater response toward CO in relation to other gases at room temperature at an applied voltage of 2 V. The response to 50 ppm CO gas was 1.48. Catalytically active Au NPs can enhance the number of chemisorbed oxygen ions when Au NPs are well-dispersed onto the WS_2_ surface, leading to further reactions among the CO molecules and chemisorbed species and an increase in the detection activities in terms of spillover effects. Additionally, Schottky barriers developed at the interfaces between Au and WS_2_, which acted as a probable source of resistance modulation in the presence of CO gas. An optimum amount of Au loading was necessary to achieve the maximum response to CO gas. A small amount of Au functionalization led to a deficiency in the number of Au NPs involved in the gas detection process, whereas high amounts of Au decreased the number of sites available for target gas adsorption. When the quantity of Au was less than the optimum value (irradiated for 15 s), the gas sensor resistance increased with an increase in Au quantity because of the development of further Schottky barriers between Au and WS_2_, and more electrons were consequently moved from WS_2_ to Au NPs. However, the Au NPs were partially interconnected when the Au quantity was greater than the optimum value, which considerably decreased the baseline sensor resistance, and the number of adsorption sites accessible for CO was also decreased, resulting in a low CO response. 

**Figure 17:**
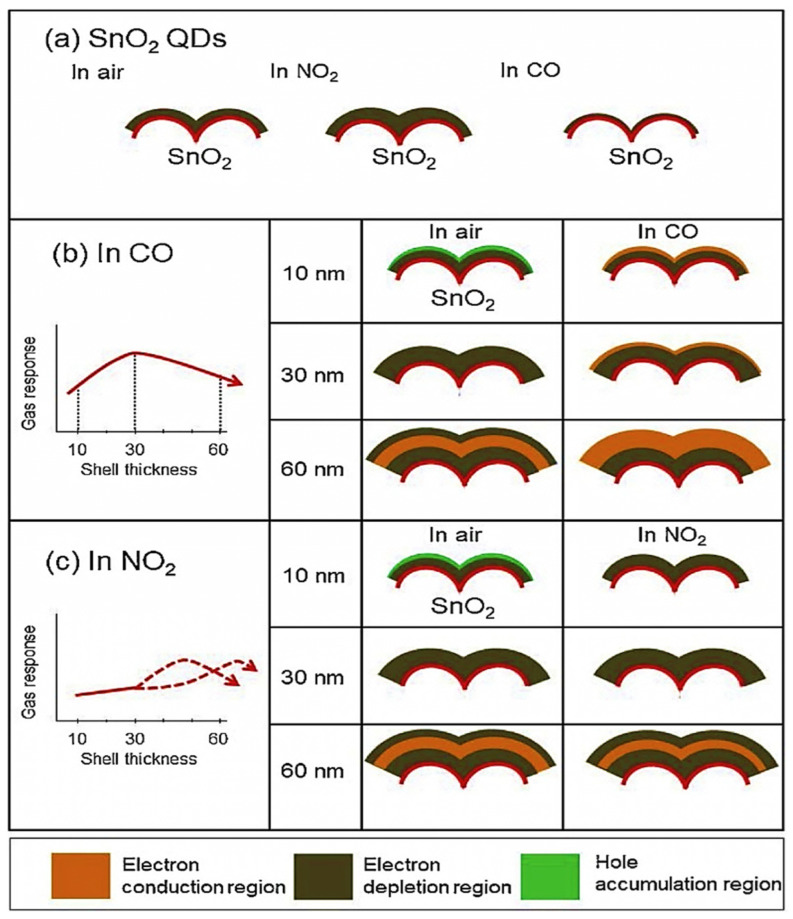
Schematic of the NO_2_ and CO-sensing mechanism for (**a**) pristine SnO_2_ QDs and (**b**,**c**) TiO_2_-layer-modified SnO_2_ QDs with different layer thicknesses. Reproduced from [108] with the permission of Elsevier.

In another study, Lee et al. [108] reported the CO and NO_2_ gas sensitivities of SnO_2_ quantum dots (QDs) with TiO_2_ layers in the self-heating mode. The TiO_2_ layer was deposited via ALD by controlling the number of ALD cycles, where the TiO_2_ layer thickness was set to 10, 30, and 60 nm to determine the impact of shell thickness on the gas response of the sensor. The sensor with a shell thickness of 30 nm showed the maximum response to CO gas (ΔR/R_a_ = 0.45 to 1 ppm CO gas at 20 V). For QD sensors with the thinnest TiO_2_ (10 nm), the TiO_2_ electrons were reduced not only by the flow of electrons to SnO_2_ but also by the adsorbed oxygen species. As a result, the entire layer of TiO_2_ became electron-depleted, and holes were also created (Figure 17a). Upon CO exposure, the electrons returned to the TiO_2_ conduction band. Moreover, the holes were initially created when the oxygen molecules were adsorbed onto the TiO_2_ surface, and the number of electrons delivered through the CO gas was utilized to neutralize the formed holes. The remaining electrons were used as charge carriers for resistance modulation. Consequently, only a part of the inserted electrons contributed to resistance modulation (Figure 17b). The TiO_2_ layer was completely depleted in air at a thickness of 30 nm (Figure 17b). The introduction of electrons through the CO gas resulted in significant resistance modulation, and the modified QD sensors exhibited the maximum response to CO gas at a thickness of 30 nm. For a large thickness of 60 nm, the initial depletion layer in ambient air was comparatively smaller than the entire layer thickness, and CO adsorption/desorption radial modulation did not result in a significant increase in the sensor response (Figure 17c). In contrast, NO_2_ sensitivity was the highest for pure SnO_2_ QDs. It was assumed that the ability of NO_2_ gas to extract electrons from the surface of SnO_2_ was higher than that of the TiO_2_ layer. Additionally, the electron transfer capability of NO_2_ gas with the SnO_2_ surface was higher than that of CO gas. For a thinner layer of TiO_2_ (10–30 nm), for which the lowest NO_2_ response was observed, the entire layer of TiO_2_ was electron-depleted upon close contact between TiO_2_ and SnO_2_. Hence, no electrons were extracted by the NO_2_ gas molecules when NO_2_ gas was present. With an increase in the thickness of TiO_2_ to 60 nm, it became partially electron-depleted, and electrons were present in the TiO_2_ layer that had to be adsorbed by NO_2_ gas molecules when NO_2_ gas was present. Any further increase in thickness decreased the sensor response, as the TiO_2_ layer was partially depleted by the injection of NO_2_. The region/volume of the initial conduction increased, and the relative change in the electron concentration of the TiO_2_ layer at the time of the introduction/removal of NO_2_ species decreased, eventually decreasing the NO_2_ response.

In another study, Kim et al. [109] investigated the CO gas-sensing characteristics of bare, Au, SnO_2_, and Au-SnO_2_-co-decorated WS_2_ nanosheets (NSs) in the self-heating mode. The Au-SnO_2_-co-decorated WS_2_ NSs exhibited a maximum response of 3.687 to 50 ppm CO gas at 4.7 V. The WS_2_ NSs possessed intrinsic resistance, leading to heat generation within the WS_2_ NSs through voltage application. Joule heating may produce heat within the WS_2_ NS grains and at the homojunctions of WS_2_-WS_2_. Furthermore, Au/SnO_2_ and Au/WS_2_ are considered as resistance sources at the interfaces between SnO_2_ and WS_2_, which are additional sources of Joule heating. For the Au-SnO_2_-co-decorated WS_2_ gas sensor, in addition to the catalytic activities of Au, the development of Schottky barriers among Au/WS_2_ and Au/SnO_2_, the heterojunctions of SnO_2_-WS_2_, and heterojunctions between Au/SnO_2_/WS_2_ afforded numerous resistance heterojunctions for the gas sensor.

**Figure 18:**
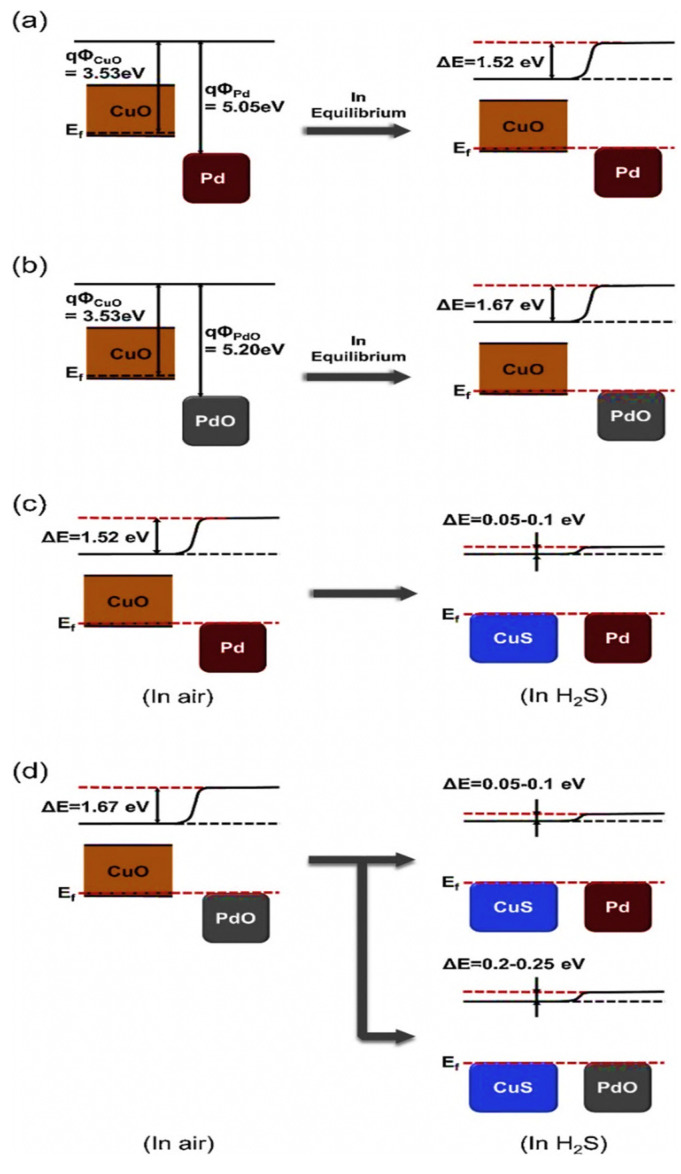
Schematics of the energy levels of (**a**) CuO/Pd and (**b**) CuO/PdO with the development of heterojunction barriers. Variations in the energy levels of (**c**) CuO/Pd and (**d**) CuO/PdO heterostructures in the presence of H_2_S gas. Reproduced from [110] with the permission of Elsevier.

In a different study, Kim et al. [110] investigated the H_2_S-sensing activities of pristine and Pd-decorated CuO NWs in the self-heating mode. In this study, a VLS method was used to synthesize CuO NWs, and subsequent Pd functionalization was performed via UV irradiation. Under the applied voltage, heat was produced due to the Joule heating effect. At 5 V, the sensor exhibited the highest response (*R_g_/R_a_* = 1.89 to 100 ppm H_2_S gas) under self-heating conditions because a significant amount of heat was produced in the gas sensor. Through CS, Pd dissociated and moved the oxygen molecules and target gases via the spillover effect. Thus, more gas molecules reached the sensor surface, resulting in a greater response. Figure 18a,b show that the electrons travel from CuO to Pd/PdO because of the lower work function of CuO in comparison to those of Pd and PdO, which lead to the formation of HAL on the CuO side.

When exposed to reducing gases, the width of HAL in CuO decreased. This indicated that the number of holes on the CuO surface decreased. As the original volume of the hole area increased due to the heterojunctions, the decrease in a similar number of holes by injecting the reduction gas resulted in a low response to reducing gases. In the case of H_2_S gas, in addition to the modification of the original resistance induced by the heterojunction, an alternative mechanism was speculated to impact the sensing. When exposed to H_2_S, CuO was converted into CuS with a conductivity similar to that of the metal, affording a strong resistance modulation within the heterojunctions. As shown in Figure 18c,d, the potential barriers of the CuO/Pd and CuO/PdO heterojunctions are destroyed upon the transformation of CuO to CuS. Consequently, fewer electrons intersect from CuS to Pd than from CuO, which results in a higher concentration of electrons in the detection layer (CuO/CuS) than in the pure detection layer.

**Figure 19:**
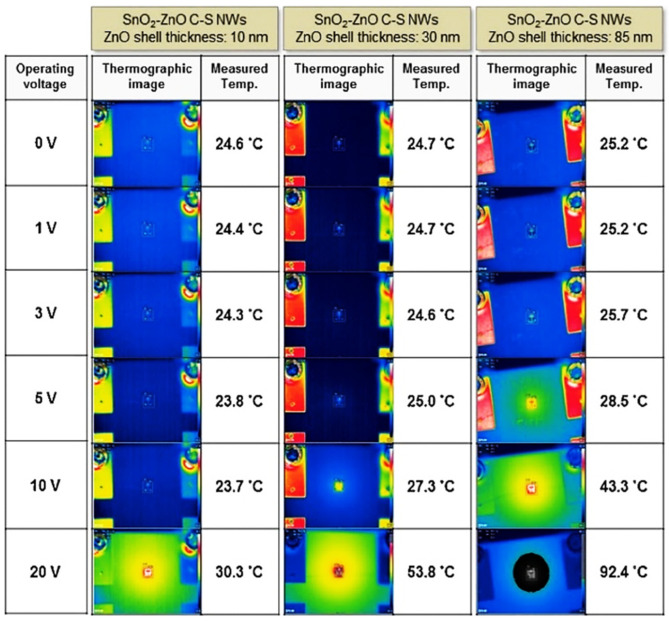
Thermographs of the Pt/SnO_2_-ZnO C-S NW sensor with various shell thicknesses at different applied voltages. Reproduced from [112] with the permission of Elsevier.

Kim et al. [111] described the CO and NO_2_-sensing capacities of pure and Au-functionalized ZnO NWs in the self-heating mode. The electron–electron collisions, heterojunctions, grain boundaries, and homojunctions acted as different heat-generating sources. The sensor fabricated with the Au-functionalized ZnO NWs showed a maximum response of 2.87 toward 10 ppm CO gas at 7 V (applied voltage) and afforded a significantly higher response in comparison to that of pure ZnO NWs. Heat dissipation into the substrate or contacts decreased the efficiency of self-heating for voltages above 7 V. As a result, a decrease in the sensor response at higher applied voltages (>7 V) was related to the high power dissipation to the substrate and a loss of sensor response. Schottky barriers developed at the Au/ZnO interfaces because of the different work functions of Au and ZnO. In addition, Au showed excellent catalytic capability for gases and efficiently adsorbed the target gases on its surfaces and transported these to the sensing material surfaces via the spillover mechanism. Consequently, more gas was adsorbed onto the ZnO NW surface in the case of sensors based on Au-ZnO NWs, leading to a high resistance modulation. Kim et al. [112] fabricated Pt-functionalized SnO_2_-ZnO C-S NWs for C_7_H_8_ gas sensing under self-heating conditions. The optimal gas sensor showed a response of 3 to 50 ppm C_7_H_8_ gas under 20 V applied voltage. The thermographs of C-S NWs are shown in Figure 19. The temperature increased with an increase in shell thickness from 10 to 85 nm. Negligible self-heating was observed for the 10 nm-thick shell sensors based on C-S NWs, whereas it was relatively apparent for a shell thickness of 85 nm. Pt NPs were more effective for C_7_H_8_ adsorption and dissociation than other reducing gases. Additionally, the methyl group of C_7_H_8_, through which it adsorbed onto the surface, contributed toward improved adsorption onto the surface. To modulate electron depletion in C-S NWs, electrical transport could occur in both the shell and regions of the core near the shell (smearing effect) when the shell thickness was less than the λ_D_. For the sensors with a 10 nm-thick ZnO layer, the complete layer of the ZnO region was electron-depleted with the possibility of generating a SnO_2_ depletion region near the interfaces. In addition, via the adsorption of C_7_H_8_ gas molecules, it was expected that the free electrons introduced by the C_7_H_8_ gas could drift through the ZnO-depleted region with no carriers, across the ZnO/SnO_2_ heterojunction, and move into the electron depletion region of the SnO_2_ core. 

**Figure 20:**
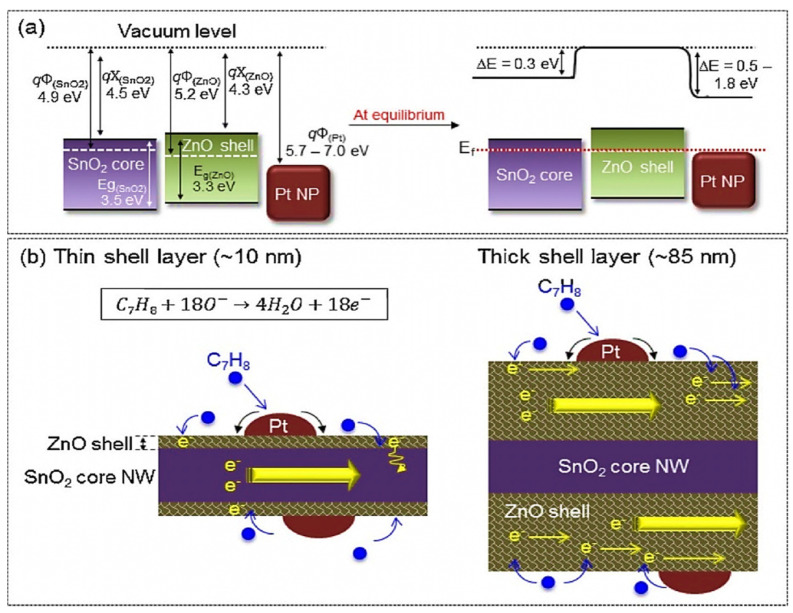
Schematic of C_6_H_6_ gas-sensing mechanisms of Pt-functionalized SnO_2_-ZnO C-S NWs. (**a**) Energy band diagrams and (**b**) Pt catalytic effect on different shell thicknesses. Reproduced from [112] with the permission of Elsevier.

There were no electronic barriers between ZnO and SnO_2_ (Figure 20a). As electric current passed primarily through the SnO_2_ core with a diameter of <50 nm, the resistance changed accordingly. In contrast, for an 85 nm-thick ZnO layer, a substantial depth of the non-depleted region existed in the ZnO shell despite the depletion region created by chemisorbed oxygen. As a result, the free electrons introduced by the C_7_H_8_ gas could drift into the ZnO shell layer via the adsorption of C_7_H_8_ molecules, thereby decreasing the thickness or increasing the concentration of electrons. Meanwhile, the main conduction volume was expected to change from the ZnO shell to the SnO_2_ core with an increase in the shell thickness. The decrease in the thickness of the ZnO shell did not decrease the initial resistance by decreasing the conduction volume and increasing the sensor response.

Furthermore, Pt NPs display catalytic characteristics through the spillover effect, which allow the adsorption of C_7_H_8_ molecules and the transfer of gas molecules to the adjacent ZnO surface (Figure 20b). For NWs with thick shells, the main current passed through the ZnO shell, which was in the vicinity of the surface and Pt NPs. Thus, Pt significantly enhanced the sensing activities. In addition, for thin-shell NWs, the main current flowed across the SnO_2_ core, which was away from the surface and Pt NPs. Consequently, Pt catalysts did not significantly improve the sensing performance.

**Figure 21:**
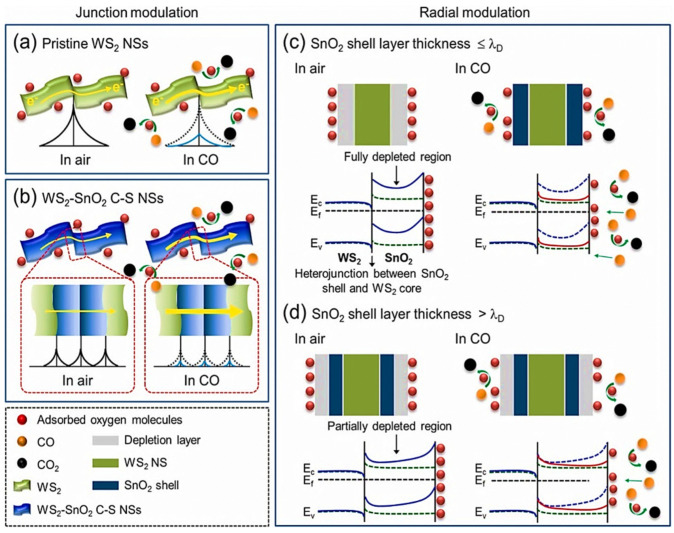
Schematic of the modulation of homojunction in (**a**) pure WS_2_ NSs and (**b**) WS_2_-SnO_2_ C-S NSs. Radial modulation in the sensors with shell thicknesses of (**c**) <λ_D_ and (**d**) > λ_D_. Reproduced from [114] with the permission of Elsevier.

As a highly toxic gas, CO does not have odor, taste, or color [113]. Therefore, the design of sensitive CO gas sensors is important to prevent the hazardous effects of CO gas on the human body. Kim et al. [114] performed CO gas-sensing studies of WS_2_-SnO_2_ C-S NSs, where SnO_2_ shells with various thicknesses (0–30 nm) were deposited on WS_2_ by varying the number of ALD cycles. The sensor designed with a 15 nm-thick shell was capable of detecting CO gas at 3.4 V under self-heating conditions. For NO_2_ gas detection, although NO_2_ sensitivity increased with an increase in the shell thickness (up to 1.4), it was very low in relation to the CO sensitivity. For a 30 nm-thick shell layer, the response (*R_a_/R_g_* = 1.4 to 50 ppm CO gas) was not different from that of the pristine WS_2_ (*R_g_/R_a_* = 1.3). The pristine sensor had numerous homojunctions, which were attributed to the sensing signal (Figure 21a). For C-S NSs, other mechanisms were observed in addition to the modulation of resistance at the homojunctions (Figure 21b) because of the different work functions of the WS_2_ core and SnO_2_ shell. As a result, both sides were electron-depleted in the case of SnO_2_ shell; one side was electron-depleted because of exposure to air and the other side was depleted due to electron flow toward WS_2_. Based on the shell thickness, the thickness of the electron depletion layer changed relative to the entire SnO_2_ shell thickness. When the shell thickness was ≤ λ_D_ of SnO_2_, the entire shell was depleted of electrons when exposed to air. Upon CO exposure, the electrons returned to the gas sensor surface, and considerable resistance modulation occurred, which resulted in a high CO response (Figure 21c). However, for shells thicker than 15 nm, only a fraction of the shell layer became electron-depleted (Figure 21d). When more electrons were accessible in the shell layer, a larger number of electrons were removed by the NO_2_ gas. This led to a high sensing signal for NO_2_ gas. As a result, only a fraction of the shell layer was electron-depleted when the shell was thicker. For a sensor with a 30 nm-thick shell, the highest response was observed to NO_2_ gas.

Kim et al. [115] fabricated an innovative self-heated CO gas sensor using a Au-functionalized network of SnO_2_-ZnO C-S NWs. The thicknesses of the ZnO shells deposited using the ALD technique were 10, 30, and 80 nm. The sensor with a shell of 80 nm-thick showed the highest response of 1.7 to 50 ppm CO gas under 20 V applied voltage. 

**Figure 22:**
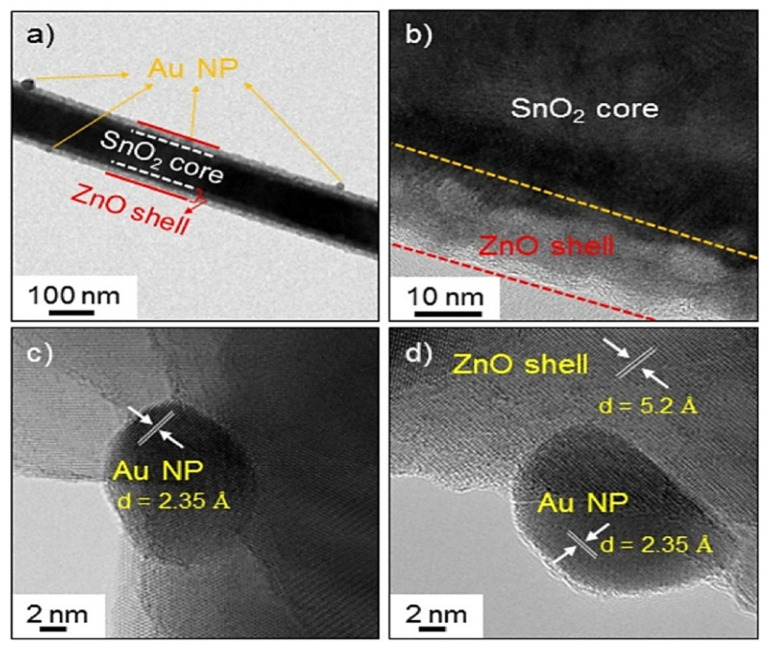
(**a**) TEM images of Au/SnO_2_-ZnO C-S NW, (**b**) HR-TEM image of SnO_2_-ZnO C-S NW, and (**c**,**d**) the corresponding HR-TEM images. Reproduced from [115] with the permission of Elsevier.

**Figure 23:**
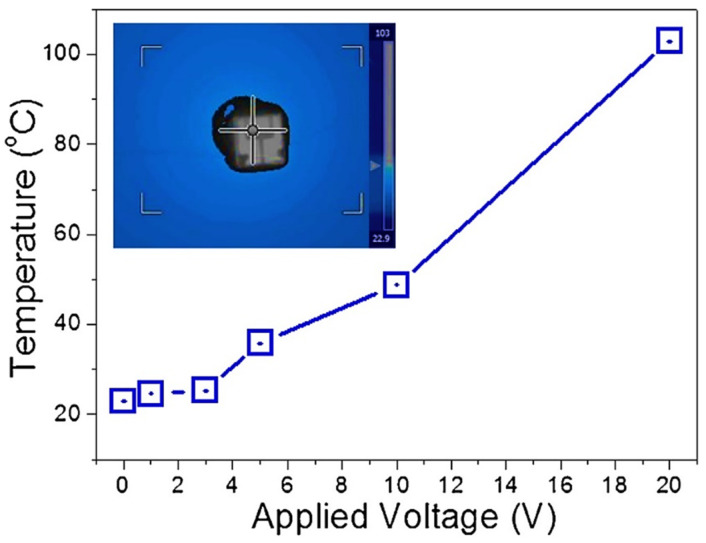
Variation in the generated temperature of the SnO_2_-ZnO C-S NW sensor with 80 nm-thick shell of ZnO as a function of the voltage applied (inset: thermograph of the sensor at 20 V). Reproduced from [115] with the permission of Elsevier.

The TEM images of a Au-functionalized network of SnO_2_-ZnO C-S NWs are shown in Figure 22, indicating the successful formation of the expected products. As shown in Figure 23, the temperature increases with an increase in the applied voltage. Owing to the different SnO_2_ and ZnO work functions, some ZnO electrons in the vicinity of the interfaces moved toward the SnO_2_ side. In addition, Au NP-functionalization introduced two features: the development of Au/ZnO nano-Schottky junctions and chemical effects of Au. In addition, the Au NPs catalytically dissociated molecular oxygen species and accordingly enhanced the reaction rate between oxygen and CO gas.

**Figure 24:**
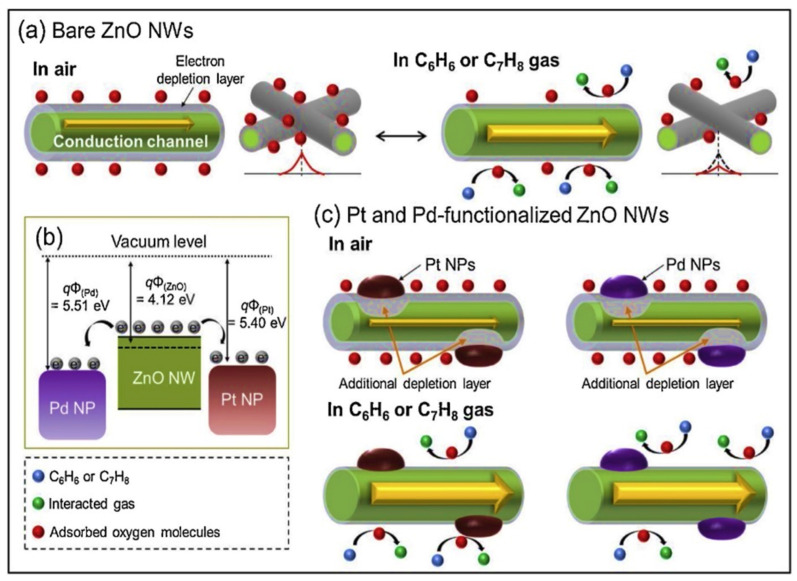
(**a**) Sensing mechanism of pristine ZnO NWs, (**b**) work functions of ZnO, Pd, and Pt/Pd-functionalized ZnO NWs, and (**c**) sensing mechanism of Pt- and Pd-functionalized ZnO NWs. Reproduced from [117] with the permission of Elsevier.

Benzene and toluene are toxic volatile organic compounds (VOCs). Benzene is a carcinogen, and its high level of exposure affects the blood, which may cause leukemia. Additionally, toluene may have an adverse effect on the nervous system, such as disrupting the brain function and impairing the ability to see, hear, and speak. It can also damage the kidneys and liver. From a medical perspective, toluene is considered a biomarker for lung cancer; unusually high toluene levels (10–100 ppb) in exhaled breath may be a sign of lung cancer. Benzene is an inert gas, and benzene rings generally exhibit low reactivity. Toluene is slightly more reactive than benzene owing to its electron-donating methyl (–*CH_3_*) group. Consequently, the development of selective gas sensors for detecting benzene and toluene gases is important [116]. Kim et al. [117] investigated the VOC-sensing properties of Pt- and Pd-functionalized ZnO NWs in the self-heating mode. The thickness of the initial sputtered metal layer (5 and 10 nm) and annealing temperature (500–750 °C) were varied to optimize Pt NP formation, while the UV irradiation time was modulated to obtain isolated Pd NPs on the ZnO NW surface. The sensors decorated with Pd and Pt showed an enhanced response to benzene and toluene gases, respectively. The response of the Pd-decorated gas sensor to 50 ppm C_6_H_6_ was 2.3 under 20 V applied voltage, and the response of the Pt-decorated gas sensor to 50 ppm C_7_H_8_ was 2.2 under 20 V applied voltage. The sensing mechanism is shown in Figure 24. For toluene sensing with Pt/ZnO, toluene is initially chemisorbed onto the surface sites of Pt/ZnO and reacts with the chemisorbed oxygen to form benzaldehyde, which is then transformed into benzoate species. The increased decomposition of benzoate species is a major contributor to the catalytic activity of Pt during toluene oxidation. Thereafter, the benzoate species were broken down into carboxylates and carboxylic acids, which were then transformed into anhydrides and carboxylates.

Anhydrides were further converted into adsorbed H_2_O and CO species, and the latter could react with adsorbed oxygen to produce CO_2_. The presence of -*CH_3_* plays an important role in the detection of toluene, as toluene can adsorb on the surface through this group [118]. Kim et al. [119] reported the H_2_S gas-sensing properties of CuO-decorated SnO_2_-ZnO C-S NWs with different shell thicknesses in the self-heating mode. The sensor with an 80 nm-thick shell showed better detection capability than sensors based on thin shells. The maximum response was 1.8 to 10 ppm H_2_S under 5V applied voltage. In addition to the development of various heterojunctions between SnO_2_-ZnO and ZnO-CuO, together with the ZnO-ZnO homojunction, the transformation of CuO to CuS should also be considered. Upon H_2_S exposure, CuO reacted with H_2_S to form an intermetallic CuS compound with a conductivity similar to that of the metal. In this manner, the *p-n* heterojunction was destroyed, and the sensor resistance was decreased significantly. For a ZnO shell thickness of 80 nm in the C–S NWs, a substantial depth of the non-depleted region was observed in the ZnO shell even though the depletion region was formed due to oxygen chemisorption. Upon H_2_S introduction, CuO was transformed into CuS by destruction of the ZnO-CuO heterojunctions, and the sensor resistance decreased significantly. A low response was observed for the sensor based on pristine SnO_2_ NWs, which was attributed to the low self-heating effect of the pure SnO_2_ gas sensor.

## 4. Irradiated Gas Sensors

**Figure 25:**
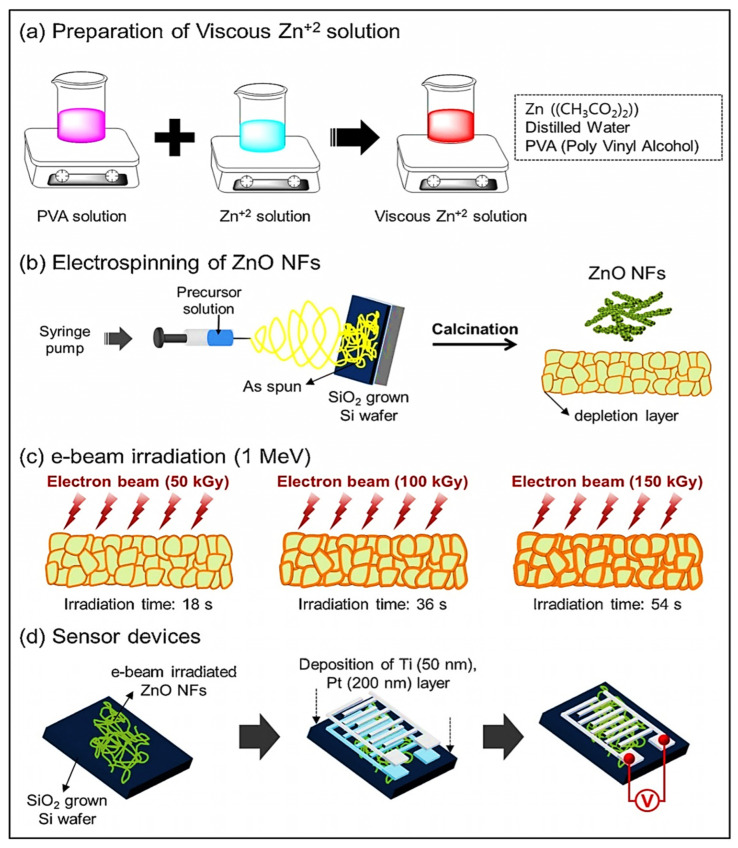
Illustration of (**a**–**b**) the synthesis of ZnO NFs, (**c**) e-beam irradiation, and (**d**) sensor fabrication. Reproduced from [127] with the permission of Elsevier.

There are various approaches to improve the gas detection properties of the sensing materials. One of the post-treatment (change in morphology or chemical composition after synthesis) strategies involves the use of low- to high-energy irradiation techniques such as ion-beam irradiation [120], laser irradiation [121], gamma ray irradiation [122], electron-beam (e-beam) irradiation, and UV light irradiation [123]. The research groups of Professors Hyoun Woo Kim and Sang Sub Kim applied high-energy irradiation to the sensing materials. The application of high-energy irradiation is promising as it can change the physicochemical properties of the sensing materials. When high-energy beams interact with the sensing material, they can modify the molecular and structural properties of the materials, including the ionization and development of various types of defects such as interstitial atoms and vacancies [124]. In this manner, irradiation modifies the structure of the sensing material and may therefore alter its detection capability. The degree of modification is determined by the radiation energy, radiation dose, and material properties [125]. Among various irradiation techniques, e-beam irradiation is one of the common irradiation techniques that may induce defects in the sensing materials [126]. Kim et al. [127] designed highly sensitive ZnO NFs for H_2_ sensing via e-beam irradiation (1 M*eV*) and investigated the effect of e-beam doses (50, 100, and 150 kGy) on the sensing characteristics. Figure 25 shows the schematics of the synthesis, irradiation, and sensor fabrication processes of the ZnO NFs. 

**Figure 26:**
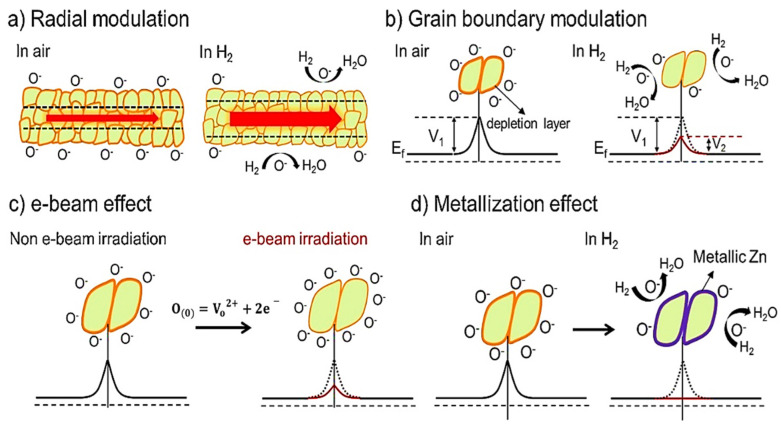
Schematic of the H_2_-sensing mechanism of the e-beam-irradiated ZnO NF sensor. (**a**) Radial modulation, (**b**) grain boundary modulation, (**c**) effect of e-beam, and (**d**) effect of ZnO metallization. Reproduced from [127] with the permission of Elsevier.

The sensor fabricated using 50 kGy irradiation exhibited sensing properties comparable to those of the unirradiated sensor, as the e-beam dose was inadequate to generate structural defects in the sensor. In contrast, the sensors irradiated at 100 and 150 kGy afforded excellent sensing properties. The highest response was observed for the 150 kGy irradiated gas sensor with a value of 150 to 10 ppm H_2_ gas at 350 °C. The gas detection mechanism of ZnO NFs was associated with radial modulation, grain boundary modulation, metallization effect of ZnO (reduction of ZnO to metallic Zn), and radiation-induced oxygen defects (Figure 26a–d). In particular, the high responses to the sensors irradiated at 100 kGy and 150 kGy were attributed to the development of defects, as indicated by the photoluminescence (PL) studies. The irradiated sensor with a high e-beam dose exhibited higher levels of structural defects with oxygen vacancies. Oxygen vacancies acted as favorable adsorption sites for oxygen molecules, leading to more reactions between the adsorbed oxygen species and incoming H_2_ gas molecules.

Kim et al. [128] reported the H_2_-sensing characteristics of Pd-loaded ZnO NFs with respect to e-beam irradiation, where the optimized sensor irradiated at 150 kGy exhibited an excellent response of 74.7 toward 100 ppb H_2_ at 350 °C. Pd acted as a strong catalyst for oxygen molecules, resulting in a rapid and greater removal of electrons from the ZnO surface through oxygen species. Additionally, hydrogen molecules were divided into hydrogen atoms and shifted toward the ZnO surface (through the “spillover” effect), where they interacted strongly with the previously adsorbed oxygen species, thereby releasing the electrons back to the sensor surface. In ambient air, oxygen interacted with the trapped electrons at the Pd/ZnO heterojunctions. In an H_2_ environment, Pd NPs could absorb atomic hydrogen to form PdH_x_. The work function of PdH*_x_* may be sufficiently high to accept other ZnO electrons and decrease the original number of electrons in ZnO, thereby increasing the sensor response. In addition, Pd NPs could act as electron scattering points, and the variation in electron scattering produced by hydrogen adsorption resulted in additional changes in resistance. Thus, Pd-loaded sensors exhibited higher responses relative to the pristine ZnO NF sensor due to Pd effects.

Kwon et al. [129] enhanced the NO_2_-sensing properties of reduced graphene oxide (rGO) by e-beam irradiation (100 and 500 kGy). The sensor irradiated with a higher dose showed a higher response, decreased response time, and increased recovery time due to enhanced NO_2_ adsorption through defect formation and functional oxygen groups. The observed structural defects and functional oxygen groups were responsible for the beam-induced variations in the sensing performances. For an irradiation of 100 kGy, oxygen functional groups were created between the graphene nanosheets. The oxygen functional groups could provide additional surface adsorption sites to the target gases, resulting in higher modulation of the resistance of the e-beam-irradiated rGO films. Therefore, an improvement in the sensing performance through e-beam irradiation of 100 kGy was related to the production of oxygen functional groups. However, the d-spacing and oxygen content decreased with an increase in the irradiation dose from 100 to 500 kGy. As a result, at a high dose of 500 kGy, the quantity of generated oxygen functional groups was lower at 100 kGy. Thus, non-oxygenated defects played an important role in improving the sensing performance. Choi et al. [130] reported the effect of e-beam irradiation (0, 100, and 500 kGy) on the NO_2_-sensing features of Pd-functionalized rGO. The response of the unirradiated sensor and the sensor irradiated at doses of 100 and 500 kGy to 10 ppm NO_2_ were 1.027, 1.045, and 1.047, respectively. The response times of Pd-RGO, Pd-RGO-100 kGy, and Pd-RGO 500 kGy to 10 ppm NO_2_ gas were 389, 335, and 345 s, respectively. Thus, the sensor developed with the highest irradiation dose exhibited the maximum NO_2_ response. The corresponding recovery times for these sensors were 808, 766, and 816 s, respectively. For the sensor irradiated at 100 kGy, the quantity of oxygen decreased, and the amount of carbon increased with respect to the non-irradiated sensor. As a result, the improvement in the gas response was attributed to a high number of oxygen vacancies or functional oxygen groups in this sensor with additional adsorption sites for the target gas molecules. For the sensor irradiated with a higher dose, the oxygen content of rGO increased due to e-beam irradiation. These sites were capable of adsorbing large quantities of oxygenated functional groups. Moreover, non-oxygen related defects, including carbon vacancies and 5-8-5 defects, could be produced after irradiation at 500 kGy with a potential impact on the response to NO_2_.

Ion implantation is a method for injecting atoms/ions into the host solids at the desired depth of the host material, which allowed the alteration of the surface properties of the solids. This process allowed the precise placement of ions at the preferred depth of the solid as a function of energy, ion mass, and implantation angle. In contrast to other techniques, the implantation of ions for doping affords several advantages, such as excellent reproducibility and the ability to embed any atom into any solid material without limitations due to diffusion, precipitation, and segregation solid solubility. The interactions between the electrons and nuclei of the host material occur via ion implantation. At low energies, the incident ions lose their energy primarily because of the elastic collisions with solid nuclei (nuclear energy loss). The properties mainly change according to the characteristics and quantity of the incident atoms, their diffusion characteristics, and the number and types of defects. At high energies, electron excitation/ionization occurs (electronic energy loss) due to inelastic collisions [131,132,133]. Kwon et al. [134] irradiated SnO_2_ NWs with He ions (45 MeV) through different ionic fluences, where the NO_2_ response of the sensor increased considerably with an increase in the ion fluence. The highest response was achieved under an ion fluence of 1 × 10^16^ ions/cm^2^. PL studies demonstrated the generation of structural defects and/or tin interstitials upon irradiation. In addition, the ionic ratio of Sn^2+^/Sn^4+^ increased upon ion-beam irradiation, indicating the development of surface Sn interstitials. Accordingly, the increased NO_2_ response was associated with the development of surface defects, which were made up of Sn interstitials. In another study, Kim et al. [135] described the NO_2_-sensing characteristics of Sb-ion-implanted (30 keV) SnO_2_ NWs at various irradiation doses (2 × 10^13^, 2 × 10^14^, and 2 × 10^15^ ions/cm^2^). The sensor designed with the lowest dose of 2 × 10^13^ ions/cm^2^ exhibited an excellent sensing performance. It showed a high response of 118 to 1 ppm NO_2_ gas at 300 °C. Electron paramagnetic resonance (EPR) studies supported the generation of a large number of oxygen vacancies upon Sb implantation, which not only improved oxygen adsorption but also provided a large number of electrons to the SnO_2_ conduction band. Surface defects facilitated oxygen adsorption, thereby increasing the resistance following implantation. Contrarily, for high doses, the initial sensor resistance decreased because of the development of Sn interstitials, where these created a donor level within the SnO_2_ conduction band. The surface defects developed not only because of the various ionic sizes of Sb^5+^ and Sn^4+^ but also because of the high energy of the ion-implantation process. In the case of high implantation doses for which low responses were observed, more defect clusters were formed, which decreased the number of gas adsorption sites on the sensor surface. Furthermore, for the sensor developed using a low irradiation dose, more oxygen vacancies were generated, which promoted NO_2_ gas adsorption.

**Figure 27:**
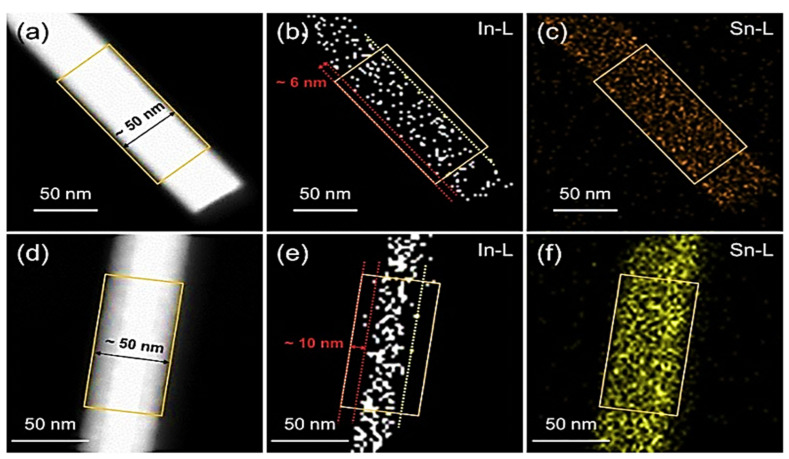
EDS color mapping images showing distribution of In and Sn elements of In-implanted SnO_2_ NWs at a dose of (**a**–**c**) 2 × 10^14^ ion/cm^2^ and (**d**–**f**) 1.8 × 10^15^ ion/cm^2^ Reproduced from [136] with the permission of Elsevier.

**Figure 28:**
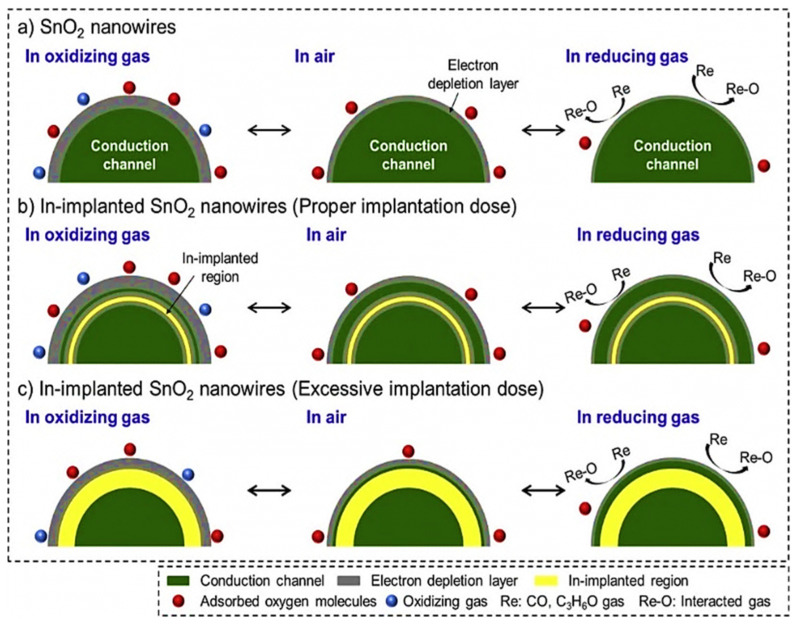
Schematic of the gas-sensing mechanisms of (**a**) SnO_2_ NWs without implantation and In-implanted SnO_2_ NWs with (**b**) optimal and (**c**) excessive implantation doses. Reproduced from [136] with the permission of Elsevier.

Kim et al. [136] fabricated SnO_2_ NWs and implanted indium ions (30 keV) using irradiation doses of 2 × 10^14^ and 1.8 × 10^15^ ion/cm^2^ for the sensing of reducing and oxidizing gases. The sensor irradiated with a lower dose showed a response of 19 to 1 ppm NO_2_ gas at 300 °C. The TEM-EDS color mapping images of the low-dose implanted SnO_2_ NW sensor are shown in Figure 27a–c. A diameter of ≈50 nm for the SnO_2_ NW was observed with In ions diffused up to a depth of ≈6 nm. The TEM-EDS color mapping images of the high-dose-implanted SnO_2_ NW sensor with an In-implanted depth of ≈10 nm is shown in Figure 27d–f. Notably, In diffused to certain depths in both cases. Consequently, a homo-core (the deeper region inside the SnO_2_ NWs)–shell (implanted region) structure was developed due to implantation. The implanted gas sensors exhibited higher responses relative to those of the pristine sensor, and the sensor fabricated with a lower dose showed the highest response among the implanted sensors. The sensing mechanism of the SnO_2_ NWs was elucidated through a homo-C-S structure (Figure 28). For sensors implanted at low doses, In^3+^ species were deeply diffused into the SnO_2_ NWs, where the conduction channel of the SnO_2_ NWs (highlighted in green) formed the core. In contrast, the In-implanted region (highlighted in yellow) formed the shell, which consisted of fewer electrons. Accordingly, the electrons traveled from the core region to the shell region, and two additional electron depletion layers were formed at the interface between the SnO_2_ conduction channel and the implanted area. Consequently, various sources of resistance modulation were established, and a significant resistance modulation was observed when exposed to NO_2_ gas. For the sensors implanted at higher doses, the electrons in the core SnO_2_ region did not flow toward the shell layer as the shell region had many free electrons; accordingly, the electron depletion layers were not formed between In^3+^ and the SnO_2_ conduction channel interfaces. Thus, there were few adsorbed oxygen ions at the sensor surface, which affected the sensor response.

## 5. Flexible Gas Sensors

Considering the types of substrates utilized in gas sensor fabrication, the gas sensors are divided into flexible and non-flexible (rigid) sensors. Non-flexible sensors are fabricated on rigid substrates. A gas sensor should be flexible in the terms of mechanical deformations and have properties that make it suitable for wear. Flexible gas sensors are fabricated on flexible substrates, and their properties should not significantly change upon titling, stretching, and bending [137]. Flexible gas sensors with low cost, low weight, high stretchability, high flexibility, and high conformability may provide a good platform for wearable gas sensors and are important for ambient atmosphere monitoring at room temperature [138,139,140]. Various flexible substrates such as polyethylene terephthalate (PET) [141], polyimide (PI) [142], and Kapton [143] can be used for the fabrication of wearable sensors. An ideal smart sensor capable of being integrated into electronics should have properties such as (i) flexibility and transparency, (ii) room temperature operation, (iii) rapid response/recovery, (iv) low detection limit, high sensitivity, low cost, and eco-friendliness [144]. 

**Figure 29:**
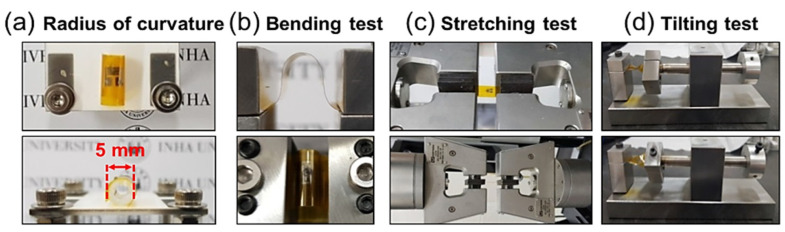
Digital photographs showing the (**a**) radius of curvature, (**b**) bending test, (**c**) stretching tests, and (**d**) tilting test. Reproduced from [107] with the permission of Elsevier.

The selection of the flexible substrate and sensing material is the main challenge faced in developing a flexible gas-sensing platform. Some sensing layers may crack or separate from the substrate when strained [145]. An overview of some of the most relevant research articles based on flexible gas sensors published by various Korean research groups in recent years is included below. Two-dimensional TMDs are among the most suitable materials for the realization of flexible sensors [146]. Kim et al. [107] reported the CO gas-sensing properties of pristine and Au-functionalized (UV-irradiated for 15 s) WS_2_ nanoflakes on flexible PI substrates under self-heating conditions. Au-decorated WS_2_ nanoflakes demonstrated outstanding CO selectivity and excellent flexibility (Figure 29).

**Figure 30:**
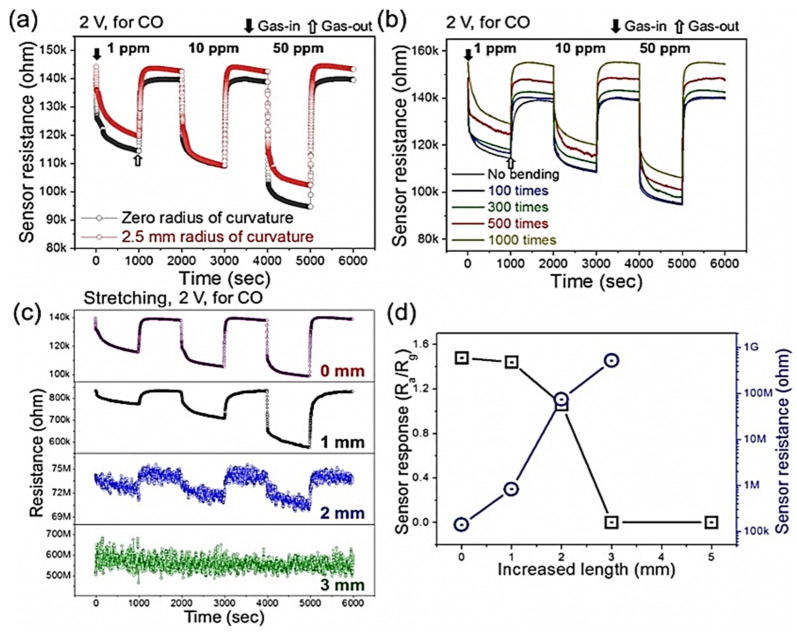
Curves showing resistance as a function of time for the sensor based on Au-functionalized WS_2_ for response toward 50 ppm CO at 2 V without bending and with bending at the radii of curvature of (**a**) 2.5 mm and (**b**) 4 mm for several bending cycles. (**c**) Resistance curves of the sensor with Au-functionalized WS_2_ nanoflakes stretched for different lengths. (**d**) Response and resistance of Au-WS_2_ gas sensor (irradiated for 15 s) with increased length (toward 50 ppm CO gas at 2 V). All the gas-sensing tests were performed at room temperature (25 °C). Reproduced from [107] with the permission of Elsevier.

Figure 30a–d show different states such as bending and stretching, in which the sensor on the PI substrate is tested under an applied voltage of 2 V for a response toward the CO gas. The sensor response was almost unaffected even after bending 1000 times. 

The gas responses at 1, 10, and 50 ppm levels of CO after bending 1000 times were 1.202, 1.290, and 1.463, respectively, indicating good flexibility, stability, and repeatability of the developed sensor. It was concluded that the sensor could be operated under bending and stretching conditions, demonstrating its high flexibility for practical applications. 

**Figure 31:**
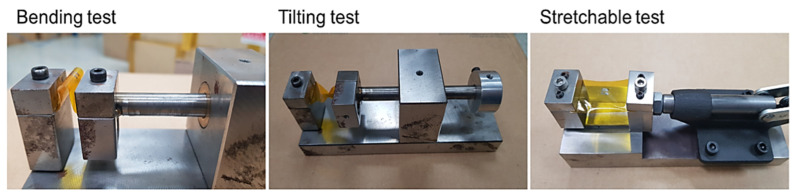
Digital photographs of the Au-SnO_2_-co-decorated WS_2_ NS sensor upon bending, tilting, and stretching. Reproduced from [109] with the permission of Elsevier.

**Figure 32:**
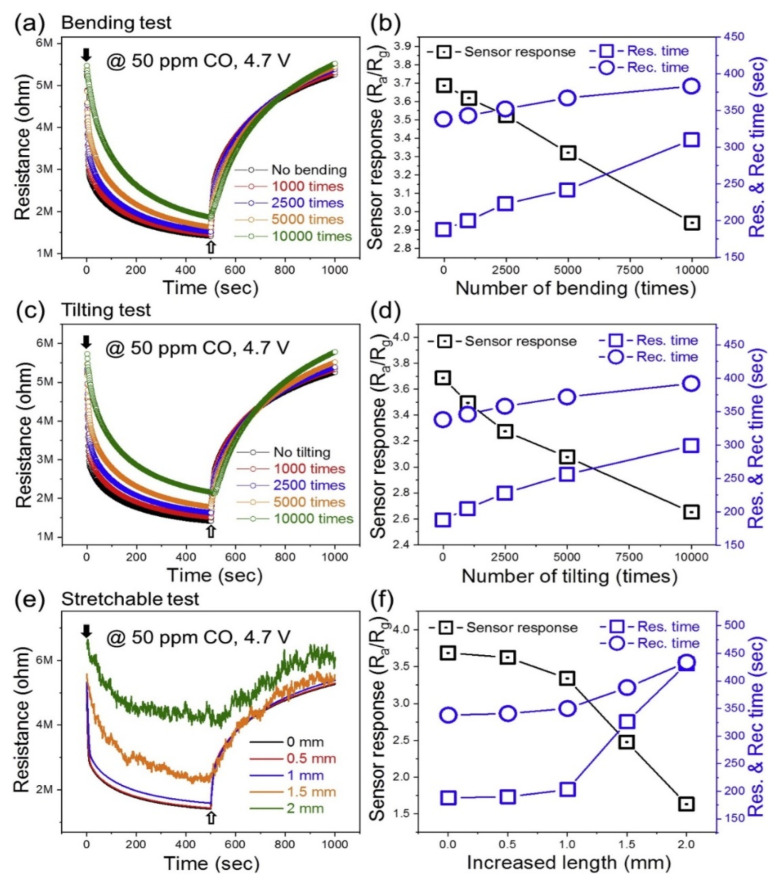
(**a**) Resistance plot of Au-SnO_2_ co-decorated WS_2_ sensor for response to 50 ppm CO gas upon 10,000 bending cycles. (**b**) Corresponding response of the sensor and response/recovery times upon variation in the number of bending cycles. (**c**) Resistance plot of the sensor based on Au-SnO_2_ co-decorated WS_2_ NS upon exposure to 50 ppm CO gas with different tilting cycles. (**d**) Corresponding response of the sensor and response/recovery time as a function of a change in the number of tilting cycles. (**e**) Resistance plots of the Au-SnO_2_ co-decorated WS_2_ sensor considering its response to 50 ppm CO gas with variation of the stretching amount. (**f**) Equivalent sensor parameters as a function of increased length. All the sensing tests were performed at room temperature (24 °C). Reproduced from [109] with the permission of Elsevier.

In a different study, Kim et al. [109] reported the CO gas-sensing properties of Au-SnO_2_-co-decorated WS_2_ nanosheets, where the maximum response of 3.687 was observed for 50 ppm CO at 4.7 V. Digital photographs of Au-SnO_2_-co-decorated WS_2_ NS sensor under bending, tilting, and stretching conditions are shown in Figure 31. Gas detection tests were performed under various mechanical conditions to confirm the flexibility of the designed gas sensor. As shown in Figure 32a–f, the optimized gas sensor shows excellent flexibility upon tilting, bending, and stretching. Even after 10,000 bending cycles, tilting 10,000 times, and stretching up to 1 mm, the performance of the gas sensor did not change, confirming the high flexibility of the sensor owing to the use of a PI substrate and the *2D* structure of the sensing layer. 

**Figure 33:**
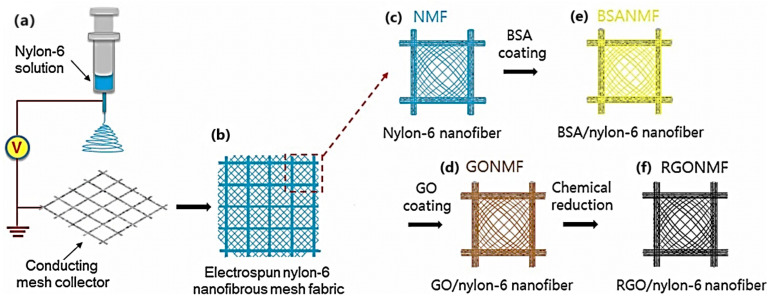
Schematic of the preparation of RGO/nylon-6 nanofiber. (**a**) electrospinning of nylon-6 solution (**b**) electrospun nylon-6 fabric (**c**) nylon-6 nanofiber (**d**) BSA-nylon-6 nanofiber (**e**) GO coating (**f**) RGO-nylon-6 nanofiber. Reproduced from [147] with the permission of Elsevier.

rGO affords various advantages such as excellent electrical conductivity, high mechanical flexibility, high chemical and thermal stability, and low weight. Park et al. [147] developed a flexible NO_2_-gas-sensor-based rGO nanofibrous mesh fabric. Figure 33 shows a schematic illustration of the synthesis. At room temperature, the absolute response values were ≈23.9% toward 8 ppm NO_2_ gas for the flat gas sensor and 26.5% for the bent RGONMF gas sensor. This result showed that the sensor performance well at an extreme radius of curvature (*R* = 1.0 mm).

**Figure 34:**
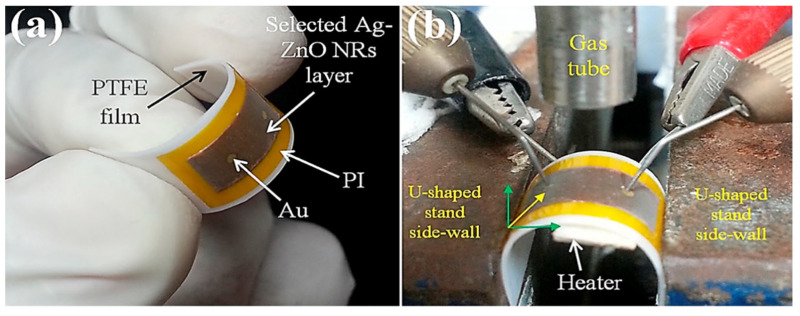
Optical images of (**a**) flexible ZnO NRs gas sensor and (**b**) experimental setup at specific bending angles. Reproduced from [150] with the permission of Elsevier.

Jang et al. [148] synthesized TiO_2_ nanotubes (TNTs) directly on titanium thin films deposited on plastic substrates. The fabricated gas sensor was used for CO-sensing applications. With a minimum bending radius of 6 mm, the gas sensor showed no electrical failure or repetitive bending deformation for up to 20 cycles, demonstrating the high flexibility and stability of the gas sensor. Yi et al. [149] prepared vertically aligned ZnO nanorods and graphene hybrid architectures for the realization of flexible and highly sensitive C_2_H_5_OH gas sensors. Repeated bending–unbending cyclic experiments confirmed that the sensor resistance changed by ≈0.2 ± 0.01% upon bending the substrate to a radius of curvature of <0.8 cm. Furthermore, sensor resistance fully recovered to its original value when the substrate returned to its release state after 100 cycles. Uddin et al. [150] fabricated a flexible C_2_H_2_ sensor for the first time (Figure 34). Ag-loaded vertical ZnO NRs (6, 8, and 10 s) were synthesized by the hydrothermal RF magnetron sputtering method.

To investigate mechanical flexibility, bending tests were performed for the fabricated sensors at various curvature angles ranging from 0° to 90° using a U-shaped support stand for different C_2_H_2_ concentrations at 200 °C. With an increase in bending, the strain between the PI substrate and sensing layer resulted in a minor change in the surface resistance. The initial response magnitudes (unbent or *θ_c_* = 0°) for 100 ppm C_2_H_2_ were 9.33, 13.8, and 9.57 for the 6, 8, and 10 s Ag-loaded sensor, respectively. The response magnitudes of all samples were retained without a significant degradation up to a bending angle of *θ_c_* = 30° (bending radius of ≈12 mm). With an increase in the bending angle, the sensor exhibited a negligible response decrease of 1.3% for *θ_c_* = 45°, 1.7% for *θ_c_* = 60°, and 2.1% for *θ_c_* = 90°, which was because of the low binding energy and low charge transfer between the strained sensing layer atoms and target gas molecules at large bending angles. Moreover, for a large bending state, the ohmic contact distance increased, resulting in a slight increase in resistance and a low drop in the response value. Kang et al. [151] developed a flexible gas sensor based on the *2D* layer assembly of Pt-ZnO NPs on rGO for NO_2_ gas sensing. To demonstrate its stable and reliable operation as a wearable sensor, the NO_2_-sensing characteristics of the sensors were investigated upon the application of external mechanical stress. Therefore, mechanical bending tests were performed directly on the flexible sensor on the PI substrate. The dynamic resistance curves obtained during 450 bending cycles were evaluated, with each cycle having a bending angle of 90°. Interestingly, a stable baseline resistance was observed with a negligible resistance drift (1.73% increase) even after 450 bending cycles. The scattered resistances in the first 50 bending cycles were mainly owing to the stabilization of the mechanical stress and partial detachment of the drop-coated sensing layer. The synthesis of perovskite oxides such as SrTiO_3_ requires long calcination at elevated temperatures, which limits their application to flexible electronics. Duy et al. [152] reported an effective laser-assisted sol–gel method to obtain SrTiO_3_ NPs in selective areas on PI substrates. The violet laser power was only 1 W but was sufficient to crystallize the material in a short period (a few seconds). The authors used it together with the CNTs to detect changes in humidity at room temperature. The sensor in the resistive mode with a lower power usage (approximately 0.2 *μ*W) was used for the long-term monitoring of humidity of ≥2% RH. The performance of the flexible sensor was maintained after 5000 bending cycles at a 1.5 cm radius.

In another study, Kim et al. [153] used micropatternable double-faced ZnO NFs for the realization of flexible gas sensors on a PI substrate with SWCNTs as the electrode. The response of the sensor to 500 ppm NO_2_ at 270 °C increased from 202.2 to 297.6 after 10,000 cycles. Moreover, the characteristic times improved. The response and recovery times decreased from 36.0 to 19.2 s and 20.3 to 11.5 s, respectively. Furthermore, the recovery increased from 89.4% to 94.8%. Overall, the characteristics of the gas sensor improved with an increase in the number of bending cycles from 0 to 10,000. However, despite an increase in the numerical values during the evaluation of sensor characteristics, the resistance gradually increased under the same conditions, with significant fluctuations. This behavior indicated the deterioration of the sensor. After flexibility tests comprising 10,000 cycles, the NRs appeared bent, indicating that a constant strain was applied to the NRs during the repetitive flexibility test. Due to repetitive strain, the breakaway of the NRs from the shells occurred, and this was the first sign of device failure. In contrast, the shells appeared to maintain their structures, and cracked shells were rarely observed. In addition, Kim et al. [154] used *2D* MoS_2_ on a plastic substrate to realize a flexible gas sensor for the detection of NO_2_ and NH_3_ gases. The flexibility of the MoS_2_-based gas sensor was evaluated after 2000, 4000, and 6000 bending cycles. The gas responses of the flexible gas sensors were maintained without any significant degradation and even showed some enhancement. This increased response at less than 4000 bending cycles was likely attributed to the formation of microcracks induced by the strain generated during the bending test. Microcracks and defects created more edge sites than those present in the initial *2D* MoS_2_, which led to increased gas adsorption.

## 6. Other Resistive-Based Gas Sensors (Glass/Silicon/MOF-Based Gas Sensors)

In addition to the above-mentioned strategies, other types of chemiresistive gas-sensing devices, such as glass, silicon, and MOF-based gas sensors, have been investigated by the Korean groups in the past few years. In the following section, some of the key research papers on glass-, silicon-, and MOF-based gas sensors are reviewed.

### 6.1. Glass Gas Sensors

Glass is a cheap material and can be modified via simple procedures. In addition, glass can be simultaneously used as a substrate and detecting layer [155]. However, there are limited studies on the use of glass as a sensing material. To employ its sensing characteristics, glass should be significantly enriched with alkalis. As the sensing response includes diffusion, the operating temperature can be decreased by the increased doping of mobile Li instead of Na. The polarization of glass is essential and should be performed prior to the sensing test. The polarization voltage can be calibrated to ensure that the accumulated charge is accurately determined prior to sensing. This allows the baseline current to be set to zero, thereby significantly increasing the resistance-based sensing response. To ensure long-term stability, it is necessary to re-polarize the glass once the basement current drifts below zero [156]. The origin of the gas-sensing mechanism is different from the mechanism used to detect metal oxides, which is attributed to the development and thickness of either an electron depletion layer or an HAL in the *n-* and *p*-type metal oxides, respectively. In the case of a soda-lime glass gas sensor, the primary detection mechanism is the development of surface impurity phases when the glass sensor is exposed to target gases [156,157]. Sophia et al. [156] reported that soda-lime glass is an active electrochemical material with significant gas detection characteristics toward various oxidizing and reducing gases such as NO_2_, C_6_H_6_, C_6_H_12_, and CO at high temperatures (300–500 °C). A chemically inert CO_2_ gas was also detected at a 1 V DC bias. 

**Figure 35:**
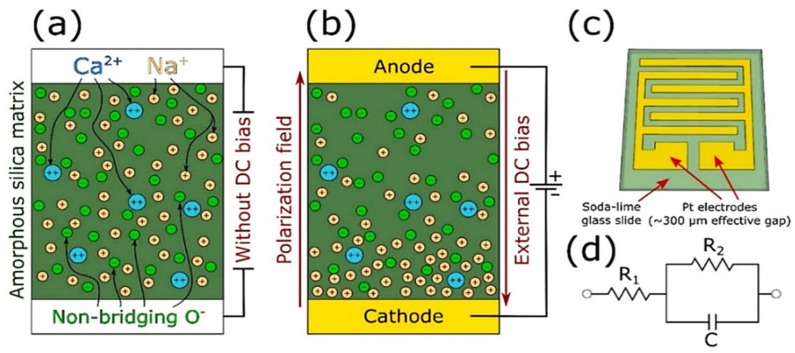
Schematic of the structures of (**a**) unbiased and (**b**) DC-biased soda-lime glass, (**c**) testing device design, and (**d**) the corresponding structure of an electrochemical cell (Randles circuit). Reproduced from [156] with the permission of Elsevier.

Alkali ions doped into the silica glass matrix were positively charged, and as a result, these were sensitive to the electric field. Alkali ions were compensated by the less mobile, negatively charged, and non-bridging oxygen units. By providing sufficient ionic mobility via thermal activation, the cations could be rearranged under an external electric field by setting a concentration gradient responsible for the macroscopic polarization of glass (Figure 35a). Once the interdigitated metal electrodes were deposited onto the soda-lime glass slides (Figure 35c), an electrochemical cell could be created where the glass slide acted as an electrolyte because of its high ionic conductivity. The corresponding schematic of an electrochemical cell (Randles circuit) constituted a parallel combination of a double-layer capacitor *C* and resistor *R*_2_, which were connected in series to resistor *R*_1_ (Figure 35d). When the soda-lime sample was electrically coupled to the DC voltage source, the external electric field induced the redistribution of Na^+^ (Figure 35b), thereby charging capacitor C. The electrochemical cell was completely charged, and the current attained its asymptotic minimum within an infinite time limit. As the target gas entered the air, its surface phase composition could be modified through the development of carbonates and/or hydroxides of the alkali elements, resulting in a change in the dielectric characteristics. Furthermore, Na_2_CO_3_ formation as a chemical reaction product between Na^+^ and atmospheric CO_2_ could passivate the charged Na^+^ species, thereby decreasing the electric charge of the polarized soda-lime glass sample because of the greater content of the reactive alkali ions near the cathode. Additional phases of impurities such as NaNO_3_ and NaOH could also form due to the reaction of glass with other gases (NO_2_ and H_2_O). Furthermore, the variation in the oxygen concentration could change the concentration of non-bridging oxygen units, which changed the concentration of compensated Na^+^ species, thereby changing the accumulated charge. Notably, both the changes in the dielectric constant and passivation of Na^+^ were attributable to the development of impurity phases, indicating the total current recorded throughout the sensing tests, which was dependent on the accumulated charge and capacitance. Similarly, the phases of impurities could also alter the conductivity of the soda-lime glass by decreasing the diffusion of Na^+^ or because of the development of free conducting electrons/holes. These effects afforded a chemiresistive gas-sensing response and the development of surface impurity phases when exposed to target gases, which was the primary reason for the gas-sensing characteristics of soda-lime glass.

**Figure 36:**
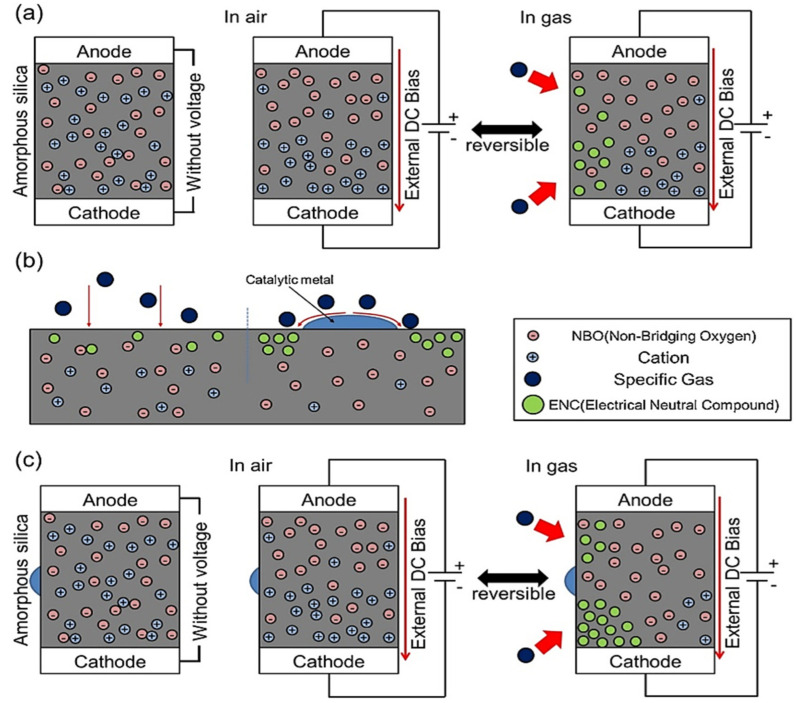
Schematic demonstrating the detection mechanism of noble-metal-functionalized soda-lime glass. (**a**) Reversible change in soda-lime glass upon the introduction and removal of target gas. (**b**) Spillover effect due to metal functionalization. (**c**) Reversible change in the metal-functionalized soda-lime glass by the introduction and removal of the target gas. Reproduced from [157] with the permission of Elsevier.

In another study, Kim et al. [157] described the gas-sensing characteristics of Pd-, Pt-, and Au-functionalized glass sensors. In this study, Pd, Pt, and Au functionalization revealed high selectivity toward C_6_H_6_, C_7_H_8_, and CO gases, respectively. The response (*I_g_/I_a_*) of Pd-, Pt-, and Au-functionalized glass sensors toward C_6_H_6_ (10 ppm), C_7_H_8_ (10 ppm), and CO (500 ppm) gases was 5, 2.5, and 1.3, respectively. A schematic of a glass sensor with various ions is shown in Figure 36a. Generally, positively charged alkali ions are doped into the silica glass matrix and electrically compensated through negatively charged non-bridging oxygens with significantly low mobility. During thermal activation that was achieved via the sensing temperature, cations could show adequate mobility and reorganize themselves under the effect of a large external electric field. This resulted in a cationic concentration gradient that was responsible for the macroscopic polarization of glass. A glass gas sensor with metallic electrodes could be considered as an electrochemical cell in which the glass slide with high ionic conductivity acted as an electrolyte. Na^+^ with high mobility were redistributed via the effect of an external electric field, thereby charging the capacitor. Then, the total current attained its minimum value. By injecting the target gas into the gas chamber, the electrical properties of the glass sensor could be favorably modified owing to changes in its surface composition due to carbonate formation and mobile cation hydroxides. Since the dielectric properties of silica glasses depend on its surface composition, they could be modified with the development of surface impurity phases. Owing to the high alkaline ion concentration near the cathode, the chemical reactions between Na^+^ and target gases formed compounds that could passivate Na^+^ species, thereby decreasing the polarization of the soda-lime glass gas sensor; this phenomenon increased the overall current. Phases of impurities, such as NaCO and NaC_x_H_y_, could develop due to the reaction of Na^+^ with CO, C_6_H_6_, and C_7_H_8_ gases. In addition, impurities could affect the conductivity of the soda-lime glass sensor by decreasing the diffusivity of Na^+^ or hampering the development of free electrons, which could result in a resistive-gas sensing response. In noble-metal-functionalized gas sensors, noble metals may act as catalysts for the decomposition of target gases (Figure 36b). Owing to the catalytic activities of noble metals, many decomposed target gases could reach the glass sensor surfaces, resulting in a high response toward a specific target gas. As each metal preferred to attract a specific gas, the metal catalyst promoted selective sensing.

Kim et al. [158] investigated a Pd-functionalized soda-lime glass sensor using machine learning algorithms to accurately identify the VOC (C_2_H_5_OH, CH_3_COCH_3_, C_6_H_6_, and C_7_H_8_) and estimate the VOC concentrations. Pd played a catalytic role in facilitating the decomposition of target gas molecules and producing the spillover effect, leading to more surface reactions. To overcome the main limitation of this type of sensor (lack of selectivity due to the one-dimensional output signal), a new approach was applied, which merged the sensor response values at different working temperatures. The responses obtained at five different temperatures (300–500 °C) that were combined into five-dimensional points were analyzed using a support vector machine. After calibration using a training dataset, the detection system could accurately identify the gas and estimate its concentration. The results showed that the sensing system afforded accurate classification (100%) and a good estimation of the concentrations of the tested gases (average error of <19% in the 1–30 ppm range). Therefore, using this approach (different temperatures and machine learning), a single resistive sensor made of glass could achieve good selectivity and accurate quantification while retaining features such as simplicity, smaller size, and lower cost compared to those of an electronic nose.

### 6.2. Si-Based Gas Sensors

A large amount of Si is present on Earth. It is highly stable, non-toxic, has high carrier mobility, and allows the use of automatized manufacturing methods. The Si NWs affords a large surface area-to-volume ratio, relative ease of fabrication, the possibility of functionalization, and environmentally friendly properties, which are beneficial for gas-sensing studies [159,160,161,162]. Mirzaei et al. [163] reported a novel top electrode configuration for vertically aligned silicon NWs synthesized via metal-assisted chemical etching (MACE) using Ag as the catalyst for H_2_ gas sensing. The sensor showed a response of 17.1 to 100 ppm H_2_ gas at 100 °C. In MACE, which is an electrodeless chemical reaction, the shapes and pathways of the noble metal NPs determine the final morphology of silicon. If the noble metal NPs form a continuous porous nanometer-scale thin film on the Si substrate, Si NWs can be successfully obtained [164]. After immersion of the Si substrate in the HF/AgNO_3_ solution, the Ag^+^ species captured electrons from Si, resulting in the Ag nucleation. As the reaction progressed, the nucleated Ag gradually grew into Ag NPs, and as Ag has a higher electronegativity than Si, Ag NPs directly captured electrons from Si and formed an accumulation of holes beneath the catalyst. Subsequently, the reduction of H_2_O_2_ occurred at the surfaces of the Ag NPs, and the holes were consumed by the oxidation of Si to silica, because of which HF was rapidly dissolved. The Ag NPs moved into the Si wafer with the dissolution of the silica layer; therefore, the depth of Ag NPs in the pits increased with an increase in the reaction time. Furthermore, during the MACE of Si, the reaction was highly anisotropic because the numbers of broken Si-Si bonds differed significantly in different directions [164,165]. The sensor exhibited a high response of 17.1 to 50 ppm H_2_ at 100 °C. The high H_2_ selectivity of the Si NW sensor was associated with the high chemical reactivity of H_2_ and the lower bond energy of H-H in comparison to those of other tested gas molecules. Furthermore, H_2_ easily decomposed at the sensing temperature.

Porous Si is a promising material for low-temperature gas-sensing applications [166]. Bang et al. [167] developed heterojunctions based on porous Si and SnO_2_ NWs for low-temperature (100 °C) H_2_S gas-sensing applications. Porous Si was obtained from *p*-Si wafers via electrochemical etching, and a VLS route was employed for the synthesis of SnO_2_ NWs. The porous Si/SnO_2_ NW sensor showed better sensing properties toward H_2_S than the porous Si sensor. The porous Si/SnO_2_ NW gas sensor showed a response of 3.2 to 50 ppm H_2_S gas at 100 °C. For heterojunctions made of porous Si/SnO_2_ NWs, the available surface area for the adsorption of H_2_S gas increased owing to the presence of SnO_2_ NWs, which eventually led to a higher sensing response. In addition, a potential barrier developed due to the transfer of electrons from SnO_2_ to Si, as *p*-Si had a higher work function than *n*-SnO_2_. Accordingly, the HAL in Si was reversed to form a hole-depletion layer (HDL), and the original sensor resistance was enhanced owing to the main sensing currents flowing through the *p*-type porous Si structures. Thus, the initial hole conduction volume in porous Si decreased. In the H_2_S gas atmosphere, the gas molecules removed holes from porous Si, and the hole resistance increased. Additionally, for an insignificant initial hole conduction volume, the decrease in the hole concentration induced by H_2_S gas led to a high response. Furthermore, crystallographic defects at the interfaces between Si and SnO_2_ were formed owing to the lattice mismatch between Si and SnO_2_. The dangling bonds and defects at the interfaces between Si and SnO_2_ afforded an improved response owing to the provision of preferred adsorption sites. The difference in the lattice constants of Si and SnO_2_ resulted in the formation of dangling bonds at the interfaces, resulting in more available adsorption sites and an improved gas response.

Lee et al. [168] investigated the H_2_-sensing properties of Pd-decorated Si nanohorns (NHs). Si-NHs were synthesized using a simple chemical etching method, which was followed by Pd decoration using a UV irradiation technique. The optimized gas sensor could detect as low as 0.1 ppm H_2_ gas. It showed a very high response of 2300 to 10 ppm H_2_ gas at 400 °C. When the sensor was exposed to air, a greater concentration of holes was observed at the surfaces of the Si-NHs. When the Si NH sensor was exposed to H_2_, it reacted with the already adsorbed oxygen ions, resulting in the release of electrons and subsequent combination with holes, decreasing the hole concentration as well as the thickness of the HAL. 

**Figure 37:**
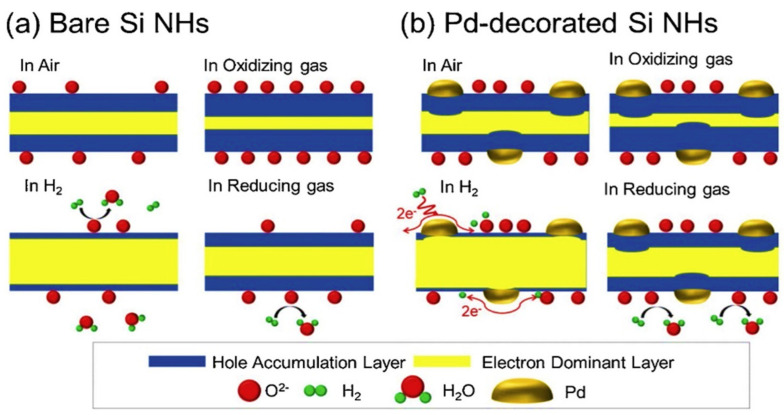
Schematics of the sensing mechanisms of (**a**) pristine Si-NHs and (**b**) Pd-decorated Si-NHs. Reproduced from [168] with the permission of Elsevier.

Figure 37a shows the sensing mechanism of the pristine Si NH gas sensor. For the Pd-decorated Si-NHs shown in Figure 37b, electrons flowed from Si to Pd upon intimate contact of Pd and Si in air. This led to the expansion of the HAL at the interfaces between Pd and Si. In H_2_ atmosphere, the released electrons resulted in the narrowing of the HAL, thereby contributing to the sensing mechanism. Furthermore, the catalytic activity of Pd NPs increased the surface reaction rates by dissociating H_2_ into active hydrogen atoms (H) and transporting atomic hydrogen to the Si NH surface. Consequently, in the presence of Pd, atomic H could easily react with chemisorbed oxygen, leading to a high response to H_2_ gas. In addition, the kinetic diameter of H_2_ molecules (2.9 Å) was very small, resulting in diffusion to the deep parts of the gas sensor. In the case of NO_2_ gas, there were insufficient electrons for adsorption by NO_2_ gas due to the formation of Pd/Si heterojunctions and the flow of electrons from Si to Pd, resulting in a low response.

### 6.3. Metal–Organic Framework (MOF)-Based Gas Sensors

MOFs are a distinct type of emergent materials that can be produced by connecting metal centers with organic linkers [169]. Owing to their unique properties, such as high surface area, ready chemical modification, structural flexibility, and tunable pore size, these have attracted significant attention for different applications [170,171,172]. In particular, MOFs have been used for chemical sensors because of their unique porous morphology, which allows the adsorption and diffusion of target gaseous molecules [173]. Drobek et al. [174] described a general approach to enhance the selectivity of NW gas sensors to H_2_ gas based on the coverage of ZnO NWs with a thin zeolitic imidazolate framework (ZIF) and ZIF-8 molecular sieve membranes. ZIF structures are a subfamily of MOFs with ultra-high porosity, structural/chemical tunability, structural flexibility, and large core surface areas. The synthetic approach involved the ALD of ZnO thin films and their successive conversion into ZIF-8 using a 2-methylimidazole/methanol solution under solvothermal conditions. The methodology was based on the use of ZnO NWs as the only zinc source, which was then used to grow a thin layer of ZIF-8 onto the NW surfaces. This method allowed the uniform coverage of ZnO NWs with a ZIF-8 selective barrier (membrane) with ultra-high specific surface area and molecular sieving characteristics. Owing to the molecular sieving effect of the thin ZIF-8 membrane, the ZIF-8-encapsulated ZnO NW sensor afforded significantly enhanced H_2_ selectivity in comparison to the sensor based on pristine ZnO NWs. The sensor showed a response of 1.44 to 50 ppm H_2_ gas at 300 °C, which was higher than the response to C_7_H_8_ (≈1) and C_6_H_6_ (≈1) gases. The large porous structures of ZIFs improved species uptake (preconcentration), and the precise pore structures of MOFs allowed molecular sieving. Both these effects likely improved the sensitivity (preconcentration effect) and selectivity (size/shape exclusion effect). Drobek et al. [175] described the synthesis of a ZnO-based sensor coated with a SIM-1 (substituted imidazolate material-1) membrane layer. SIM-1 is a material that is isostructural with ZIF-8 with a cage size of ≈0.8 nm and an expected pore aperture of <0.34 nm [176,177]. The H_2_ selective membrane of SIM-1 was used as an effective layer for molecular sieving. ZnO NWs with an optimized SIM-1 coating exhibited outstanding H_2_ selectivity at high temperatures (300 °C) when the interfering gases were present, confirming the effectiveness of these sensors in detecting H_2_. It showed a response of 2.6 to 50 ppm H_2_ gas at 300 °C, with almost no response to interfering gases. As the SIM-1 membrane did not exhibit semiconducting properties and simply acted as a molecular sieve to block the transportation of interference gases, ZnO NWs were responsible for the electron transport channels.

**Figure 38:**
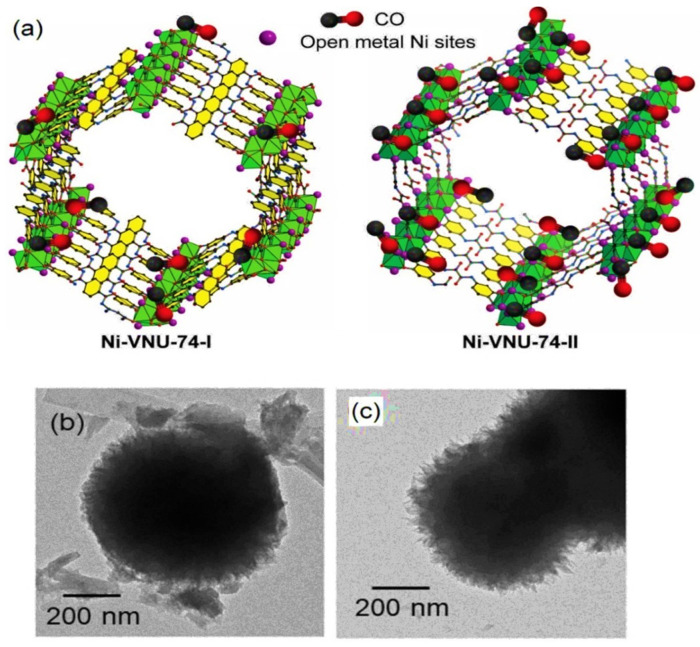
(**a**) Structures of Ni-VNU-74-I and–II. TEM images of (**b**) Ni-VNU-74-I and (**c**) Ni-VNU-74-II. Reproduced from [135] with the permission of Elsevier.

Weber et al. [178] built upon the previous studies by fabricating H_2_ gas sensors with improved sensitivity and outstanding selectivity using a combination of ZnO NWs decorated with Pd NPs and ZIF-8 as a molecular sieve. Three stages were involved in the fabrication of the desired materials: (i) growth of ZnO NWs via the VLS process, (ii) decoration of Pd by ALD, and (iii) partial solvothermal conversion of the tuned NW surface into the ZIF-8 nanomembrane. The Pd NPs improved the H_2_ sensitivity, and the ZIF-8 overcoat enhanced the sensor selectivity. The sensor showed a response value of 9 to 50 ppm H_2_ gas at 200 °C. Gases with higher kinetic diameters than ZIF-8 windows cannot pass through and reach the surface of the metal–oxide. Therefore, interfering gas molecules (all greater than H_2_) could not pass through the ZIF-8 overcoat, and no response signal was obtained from the sensor. Owing to the presence of a molecular sieve membrane, the H_2_ gas molecules reached the sensor surface. The diffusion of H_2_ by the ultra-microporous ZIF-8 network involves a thermally activated transport mechanism, which is promising for the design of a sensitive and selective high-temperature (200–300 °C) H_2_ sensor. Owing to the existence of Pd NPs, the depth of the depletion layer of electrons increased at the contact area between Pd and ZnO, which resulted in a higher resistance of the Pd/ZnO NW sensor in comparison to that of the sensor based on pure ZnO NWs. When exposed to H_2_ gas, Pd was converted into PdH_x_, and a part of the ZnO (surface) was transformed into metallic Zn. Both mechanisms afforded high conductivities, thereby contributing to the sensor response. By selecting an appropriate MOF overcoat, the pore sizes could be tailored, and this approach could also be applied to the selective sensing of other gases. ZIF-8 is a comparatively rigid but open crystalline framework with significant potential for extracting H_2_ from gas mixtures [179]. Nguyn et al. [180] investigated the CO gas-sensing characteristics of two different types of MOFs, namely Ni-VNU-74-*I* and Ni-VNU-74-*II* (Figure 38a–c), with different pore sizes and surface areas. The as-synthesized Ni-VNU-74-*I* and Ni-VNU-74-*II* exhibited high crystallinity and thermal stability, together with ultra-high specific surface areas. The gas sensor fabricated using Ni-VNU-74-*II* with an ultra-high surface area of 2350 m^2^/g exhibited the maximum response (*R_a_/R_g_* = 1.7) toward 50 ppm CO gas in comparison to other gases at a working temperature of 200 °C. The excellent sensing activity of the Ni-VNU-74-*II* sensor in comparison to that of the Ni-VNU-74-*I* sensor with a surface area of 2020 m^2^ g^−1^ was attributed to the enhanced interactions between the open Ni sites and CO gas molecules in the unique Ni-VNU-74-*II* structure with smaller pores, larger surface area, and higher concentration of Ni^2+^ sites. In addition, the observed CO selectivities of both the Ni-VNU-74-*I* and Ni-VNU-74-*II* sensors were attributed to the special structures presented in Figure 38. 

The MOFs comprised a partial charge of open metal Ni sites that could afford strong interactions with CO gas molecules. The higher density of the open metal Ni^2+^ sites resulted in a high probability of CO gas attachment. Additionally, the metal Ni^2+^ species had smaller ionic radii, and because the CO molecules were adjacent to Ni^2+^, the shorter distances of Ni^2+^_−_O allowed enhanced interactions with the oxygen atoms and CO gas molecules. Consequently, the CO-Ni^2+^ interaction increased because of the highly cooperative effects between the MOF framework and CO gas molecules. Furthermore, the smaller Ni^2+^ radius facilitated the release of its electrons owing to the interaction with the target gases, particularly CO gas. An MOF structure with an adequate pore size and volume should be selected when considering the kinetic properties of sensors such as response/recovery times, mostly when the MOF material is directly utilized as a sensing material rather than as a sieve. Lee et al. [181] investigated the NO_2_-sensing activities of two unique series of Mg-incorporated MOFs, which were labeled as Mg-MOF-*I* and Mg-MOF-*II*. The as-synthesized iso-reticular type of Mg-MOFs afforded excellent crystallinity, good thermal stability, and needle-shaped surface morphology with ultra-high surface area, which were advantageous for gas detection applications. The higher response (*R_a_/R_g_* = 1.4 to 50 ppm NO_2_ at 200 °C) observed for the Mg-MOF-*II* sensor (2900 m^2^/g) than that for Mg-MOF-*I* sensor (2520 m^2^/g) was mainly associated with the higher surface area. A higher surface area resulted in a greater number of adsorption sites, which supported the adsorption of more target gases on the gas sensor surface, leading to a greater resistance modulation and a higher sensor response. In addition, the base resistance of the Mg-MOF-*II* sensor was lower because of the enhanced adsorption of oxygen gas molecules in air. Furthermore, the Mg-MOF-*II* sensor with a larger pore size and volume showed faster response and recovery times, indicating the importance of the porosity size on the kinetic characteristics of the sensors based on the MOFs. Furthermore, MOF-derived ZnO-CuO [182] and ZnO-Co_3_O_4_ [183] nanocomposites with high surface areas resulting from MOFs were reported recently for enhanced sensing of H_2_S and C_2_H_5_OH gases, respectively. 

Finally, we have compared the gas-sensing performances of different types of gas sensors described above with research papers published by other research groups from Korea [184,185,186,187,188,189,190,191,192,193,194,195,196,197,198,199,200,201,202,203,204,205,206,207,208,209,210,211,212,213,214,215,216,217,218,219,220,221,222] in Table 3. 

## 7. Conclusions 

In this review, recent research activities of the research labs of Professors Hyoun Woo Kim and Sang Sub Kim in Korea relating the synthesis and chemiresistive gas-sensing applications of oxide- and polymer-based nanomaterials are described. In particular, this review was divided into two parts. In the Introduction section, a general viewpoint on the chemiresistive gas sensors and their limitations were described. The second section provided an overview of the different strategies for optimizing the gas detection performance, including C-S, self-heated, irradiated, flexible, Si-based, glass, and MOF-based sensors. ALD is a useful technique for suitably coating the core with a shell material with an expected shell thickness, and C-S structures with optimized shell thickness can enhance the sensing performance compared to the use of conventional nanostructures. Different *n-n* and *p-n* metal oxides can be used as either core or shell materials. One of the main challenges in the development of sensing systems is the realization of low-temperature gas sensors with a low energy consumption. This implies the complete integration of these devices with conventional electronic technologies to produce low-cost sensing devices. The self-heating gas-sensing approach has been successfully employed to overcome the high power consumption of the gas sensors. Accordingly, both *1D* and *2D* morphologies of the C-S structures have been used for self-heating studies. In addition to the metal oxides, metal sulfides, such as WS_2_, with high flexibility owing to their *2D* structures are highly promising for the realization of flexible gas sensors that can operate at low working temperatures. Another effective strategy to improve the detection performance of gas sensors is the irradiation of gas sensors with high-energy beams, such as gamma rays and e-beams. However, it is important to optimize the radiation dose to achieve the best detection properties. Moreover, glass-based, Si-based, and MOF-based gas sensors have their own advantages such as good selectivity. Based on the results of the different strategies discussed in this review, it is speculated that the gas sensors with low power consumption, high response, and high selectivity can be achieved by combining different strategies and approaches with a reasonable selection of the sensing materials.

Although many approaches have been proposed, the response of chemiresistive sensors to very low gas concentrations are not sufficiently high because factors such as temperature and humidity can affect the sensing response. Accordingly, increased research efforts have been directed toward the development of more advanced sensing materials to improve the chemiresistive performance. In particular, the directional control of material morphology and selective gas detection, with long-term stability, will continue to be a key aspect of future research.

## Figures and Tables

**Table 1:** Milestones in history of resistive-based gas sensors.

Year	Milestone	Ref.
1953	Brattain and Bardeen reported the effects of gases on the electrical conductivity of Ge-based devices	[10]
1954	Heiland reported a change in the electrical properties of ZnO in the presence of various gases	[11]
1962	Seiyama et al. reported the first resistive-based gas sensor using ZnO	[12]
1962	Taguchi patented the first SnO_2_ gas sensor	[13]
1963	Taguchi investigated effect of noble metals on the gas-sensing properties of SnO_2_	[14]
1968	Taguchi commercialized the first resistive-based gas sensors using SnO_2_	[15]
2003	Salehi reported the first self-heated gas sensor based on SnO_2_	[16]
2007	Schedin et al. for the first time reported the gas-sensing properties of graphene	[17]

**Table 2:** A comparison of the most commonly used metal oxides, with different surface morphologies, as chemiresistive gas sensors.

Dimensions	Sensing Material	Target Gas	Gas Conc. (ppm)	T (°C)	Response (*R_a_/R_g_*) or (*R_g_/R_a_*) or [(*R_a_* − *R_g_*)/*R_a_*]*100%	Res (s)/Rec (s)	LDL (ppm)	Ref.
0D	Fe_2_O_3_ NPs	CH_3_COCH_3_	100	300	11.6	4/10	0.5	[19]
	In_2_O_3_ NPs	HCHO	10	280	20	4/8	NA	[20]
	ZnO NPs	Cl_2_	200	200	1278%	6/64	5	[21]
	SnO_2_ NPs	C_2_H_5_OH	250	100	30	16/25	NA	[22]
	NiO NPs	HCHO	1	230	80	≈54/≈14	NA	[23]
	WO_3_ NPs	NO_2_	100	200	34%	24/300	5	[24]
	Co_3_O_4_ NPs	CH_3_COCH_3_	100	200	8.61	43/92	0.1	[25]
	CeO_2_ NPs	H_2_S	40	RT	5.5	64/62	NA	[26]
	TiO_2_ NPs	CH_3_COCH_3_	1000	270	15.24	10/9	0.5	[27]
	CuO NPs	H_2_S	5	40	4.9	297/54	0.2	[28]
1D	SnO_2_ NWs	CO	20	RT	4	NA	NA	[29]
	ZnO NRs	CH_3_COCH_3_	100	300	32	5/15	1	[30]
	In_2_O_3_ MRs	C_2_H_5_OH	100	300	18.33	15/20	1	[31]
	Co_3_O_4_ MRs	C_2_H_5_OH	100	220	9.8	≈1/≈11	NA	[32]
	WO_3_ NFs	CH_3_COCH_3_	50	270	55.6	13/9	0.1	[33]
	ZnO NWs	C_2_H_5_OH	500	340	10.68	6/26	NA	[34]
	Fe_2_O_3_ NRs	CH_3_COCH_3_	100	280	23.5	≈1/≈3	NA	[35]
	NiO NWs	NH_3_	50	RT	0.19	36/NA	NA	[36]
	WO_3_ NWs	NO	500	300	37	63/88	50	[37]
	TiO_2_ NTs	C_7_H_8_	50	500	3	110/800	NA	[38]
	WO_3_ NWs	C_2_H_2_	200	300	58	6/7	NA	[39]
	NiO nanochains	HCHO	50	210	NA	1/10	1	[40]
	CuO NWs	n-propanol	100	190	6.2	≈2/≈7	1	[41]
	CuO NTs	CO	100	175	1.55	24/29	0.6	[42]
	V_2_O_5_ NWs	C_2_H_5_OH	1000	330	9.03	NA	NA	[43]
2D	ZnO NSs	C_2_H_2_	100	400	101.1	11/5	1	[44]
	CuO NSs	H_2_S	10 ppb	RT	1.25	234/76	10	[45]
	Co_3_O_4_ NSs	CH_3_COCH_3_	1000	111	36.5	NA	20	[46]
	NiO NSs	C_2_H_5_OH	50	240	11.15	4/7	1	[47]
	α-Fe_2_O_3_ NSs	TEA	100	300	520	NA	1	[48]
	V_2_O_5_ NSs	CH_3_COCH_3_	100	300	~3.2	25/13	5	[49]
	TiO_2_ NSs	CH_3_OH	1	100	17.46%	NA	1	[50]
	WO_3_ NSs	NO_2_	10	100	460	54/63	1	[51]
	SnO_2_ NSs	CH_3_COCH_3_	1	280	10.4	NA	0.2	[52]
	In_2_O_3_ NSs	NOx	97	RT	89.48	16/NA	0.48	[53]
3D	Co_3_O_4_ nanocubes	CH_3_COCH_3_	500	240	4.9	2/5	10	[54]
	Fe_2_O_3_ MFs	CH_3_COCH_3_	100	220	52	8/19	NA	[55]
	ZnO NFs	C_2_H_2_	200 µL/L	375	48.2	8/11	NA	[56]
	SnO_2_ nanocages	C_7_H_8_	20	250	33.4	≈3/≈6	NA	[57]
	In_2_O_3_ MSs	C_7_H_8_	50	350	85%	12/25	0.5	[58]
	NiO nanotetrahedra	HCHO	50	250	11.6	NA	NA	[59]
	CuO MSs	HCHO	100	300	3.2	26/28	NA	[60]
	WO_3_ urchin-like structures	C_2_H_5_OH	100	350	68.56	28/12	NA	[61]
	V_2_O_5_ hollow spheres	H_2_	200	RT	2.8	50/10	10	[62]
	TiO_2_ Bowl-like structure	C_8_H_10_	100	302	1.8	12/2	NA	[63]

0D: Zero-dimensional, T: Temperature, RT: Room temperature, Res/Rec: Response time and recovery time, ppm: Parts per million, LDL: Lower detection limit, CH_3_COCH_3_: Acetone, NH_3_: Ammonia, HCHO: Formaldehyde, Cl_2_: Chlorine, CH_3_OH; Methanol; C_2_H_2_: Acetylene, H_2_S: Hydrogen sulfide, H_2_: Hydrogen, NA: Not available, NPs: Nanoparticles, NWs: Nanowires, NRs: Nanorods, MRs: Microrods, NFs: Nanofibers, NTs: Nanotubes, NSs: Nanosheets, MFs: Microflowers, NFs: Nanoflowers, MSs: Microspheres.

**Table 3:** Comparison of gas detection performances of different types of gas sensors reported by different research groups in Korea.

Sensing Material	Target Gas	Gas Conc. (ppm)	T (°C)	Response (*R_a_/R_g_*) or (*R_g_/R_a_*) or [(*R_a_* − *R_g_*)/*R_a_*]*100%	Res (s)/Rec (s)	LDL (ppm)	Ref.
Core–shell (C-S) Gas Sensors							
SnO_2_-ZnO C-S NFs	NO_2_	5	300	~0.45	NA	1	[70]
ZnO-SnO_2_ C-S NWs	CO	10	300	42	9/57	1	[71]
SnO_2_-ZnO C-S NWs	NO_2_	10	300	~155	NA	1	[72]
SnO_2_-ZnO C-S NFs	CO	1	300	~48	NA	1	[73]
CuO-ZnO C-S NWs	C_6_H_6_	1	300	~6	NA	1	[74]
SnO_2_-Cu_2_O C-S NFs	CO	10	300	5	14/14	1	[75]
Pt@SnO_2_-ZnO C-S NWs	C_7_H_8_	0.1	300	279	NA	0.1	[76]
SnO_2_-ZrO_2_ C-S NWs	NO_2_	10	150	24.7	NA	NA	[78]
SnO_2_-Cu_2_O C-S NWs	C_6_H_6_	10	300	12.5	4/4	1	[79]
CuO-TiO_2_ C-S NWs	CO	10	300	~16	NA	1	[80]
CuO-ZnO C-S NFs	CO	10	300	~8	NA	0.1	[81]
SnO_2_-ZnO C-S NFs	CO	150	300	~17	NA	3	[82]
Pd@SnO_2_-ZnO C-S NWs	C_6_H_6_	0.1	300	71	33/114	0.1	[83]
Au-decorated Si NW-ZnO C-S	H_2_S	50	300	11.22	48/64	10	[84]
Au/SnO_2_ C-S NPs	CO	1000	100	~1	NA	NA	[85]
Au/SnO_2_ C-S NPs	CO	1000	200	~2.2	NA	NA	[87]
Au@Cu_2_O C-S NPs	CO	1000	200	~5.8	NA	NA	[88]
Au@Cu_2_O C-S NPs	CO	1000	250	5.67	NA	10	[89]
Au@ZnO C-S NPs	H_2_	100	300	103.9	NA	0.5	[90]
Au@NiO C-S NPs	C_2_H_5_OH	100	200	2.54	NA	2	[91]
Au@In_2_O_3_ C-S NPs	H_2_	100	300	34.38	31/600	2	[92]
AuPd_alloy_-ZnO C-S NPs	H_2_	100	300	80	36/720	NA	[94]
Au@SnO_2_ C-S NPs	C_8_H_10_	5	300	16.17	NA	NA	[97]
Pd@In_2_O_3_ yoll-shell NPs	C_2_H_5_OH	5	350	159.02	NA	NA	[98]
Au@NiO yoll-shell NPs	H_2_S	5	300	108.90	NA	1.25	[99]
ZnO-SnO_2_ C-S NWs	C_2_H_5_OH	200	400	280	NA	0.5	[184]
SnO_2_-ZnO C-S NWs	NO_2_	5	25	6.18	NA	1	[185]
Ag-Fe_2_O_3_ C-S NPs	NO_2_	4	150	3.6	280	340	[186]
ZnO-Cr_2_O_3_ C-S nanocables	TMA	5	400	17.8	NA	0.05	[187]
Ga_2_O_3_-SnO_2_ C-S NWs	C_2_H_5_OH	1000	400	66	NA	NA	[188]
Pd@ZnO C-S NPs	H_2_	100	350	22	84/468	5	[189]
ZnO-TiO_2_ C-S NRs	NO_2_	50	RT	7.50	NA	NA	[190]
TeO_2_-CuO C-S NRs	NO_2_	10	150	425%	NA	0.5	[191]
Pd@ZnO–In_2_O_3_ C-S NPs	H_2_	100	300	42	24/240	NA	[192]
Pd@N-CeO_2_ C-S nanoflatforms	H_2_	100	350	19	60/360	0.5	[193]
**Self-Heated Gas Sensors**							
Pd@SnO_2_-ZnO C-S NWs	C_6_H_6_	50	20 V (RT)	1.62	NA	0.1	[104]
Au@WS_2_ nanoflakes	CO	50	2 V (RT)	1.48	174/30	1	[107]
TiO_2_-layer-modified SnO_2_ QDs	NO_2_	1	20 V (RT)	~90%	NA	1	[108]
Au-SnO_2_-decorated WS_2_ NSs	CO	50	4.7 V (RT)	3.68	NA	0.3	[109]
Pd@CuO NWs	H_2_S	100	5 V (RT)	1.89	NA	1	[110]
Au@ZnO NWs	NO_2_	10	7 V (RT)	3.07	NA	0.1	[111]
Pt@SnO_2_–ZnO C-S NWs	C_7_H_8_	50	20 V (RT)	3.14	NA	0.1	[112]
WS_2_-SnO_2_ C-S NSs	CO	10	3.4 V	8	NA	NA	[114]
Au@SnO_2_–ZnO C-S NWs	CO	50	20 V (RT)	1.62	NA	0.1	[115]
Pt@ZnO NWs	C_7_H_8_	50	20 V (RT)	2.86	NA	NA	[117]
Pd@ZnO NWs	C_6_H_6_	50	20 V (RT)	2.20	NA	NA	[117]
CuO@SnO_2_-ZnO C-S NWs	H_2_S	10	1V (RT)	~1.9	NA	1	[119]
Pd@Si NWs	H_2_	0.5%	30 V (RT)	106%	24/590	NA	[194]
Pd@Si NWs	H_2_	1%	1.7 V (RT)	1.6%	NA	NA	[195]
Pd@C NWs	H_2_	1000	6 V (RT)	~95%	NA	10	[196]
Nanocolumnar WO_3_ thin films	NO_2_	1	5 V (RT)	~130	NA	1	[197]
**Irradiated Gas Sensors**							
ZnO NFs (e-beam, 150 kGy)	H_2_	10	350	150	23/114	0.1	[127]
Pd@ZnO NFs (e-beam, 150 kGy)	H_2_	10	350	236.82	NA	0.1	[128]
RGO (e-beam, 500 kGy)	NO_2_	50	RT	5.280	84/1592	10	[129]
Pd@RGO (e-beam, 500 kGy	NO_2_	10	RT	1.047	345/816	2	[130]
SnO_2_ NWs (1 × 10^16^ ions/cm^2^)	NO_2_	2	150	~14	292/228	NA	[134]
Sb@SnO_2_ NWs (2 × 10^13^ ion/cm^2^)	NO_2_	1	300	111.58	NA	0.05	[135]
In@SnO_2_ NWs (2 × 10^14^ ion/cm^2^)	NO_2_	1	300	~4.9	NA	0.1	[136]
SnO_2_ NWs (e-beam, 150 kGy)	NO_2_	10	NA	1.03	16/230	NA	[198]
SWCNT-Sn/SnO_2_ composites (1KW)	C_2_H_5_OH	10	RT	~6.5	NA	1	[199]
Graphene (1000 kGy)	NO_2_	100	RT	40.68	86/499	5	[200]
**Flexible Gas Sensors**							
Au@WS_2_ nanoflakes	CO	50	RT	1.48	174/30	1	[107]
Au-SnO_2_ decorated WS_2_ NSs	CO	50	RT	3.68	NA	0.3	[109]
RGO nanofibrous mesh fabrics	NO_2_	8	RT	26.5%	NA	8	[147]
Titania NTs	NH_3_	200	350	~80%	NA	NA	[148]
ZnO NRs	C_2_H_5_OH	50	300	~90	NA	10	[149]
Ag@ZnO NRs	C_2_H_2_	1000	200	27.2	62/39	3	[150]
Pt-ZnO@RGO	NO_2_	5	RT	43.28%	528/702	0.1	[151]
ZnO nanoflowers	NO_2_	500	270	218.1	~31/~14	NA	[153]
MoS_2_	NO_2_	500	RT	~300%	NA	25	[154]
CNTs/RGO flexible film	NO_2_	10	RT	20%	NA	0.5	[201]
Flexible graphene films	NO_2_	200	RT	23%	NA	NA	[202]
Flexible graphene	NO_2_	5	RT	13%	NA	1	[203]
Flexible RGO cotton yarn	NO_2_	1.25	RT	12%	NA	0.25	[204]
GO on cotton yarn	NO_2_	3	RT	65%	NA	0.15	[205]
Graphene-based electronic sheet	NO_2_	100	RT	~38%	NA	1	[206]
ZnO-decorated rGO fibers	H_2_S	20	RT	2.68%	404/275	1.5	[207]
Ti_3_C_2_Tx/Graphene fibers	NH_3_	50	RT	6.8%	NA	10	[208]
CeO_2_-CuBr	NH_3_	5	RT	68	NA	0.02	[209]
RGO-In_2_O_3_	NO_2_	0.5	150	22.3	112/175	0.5	[210]
WO_3_/MWCNTs	NO_2_	5	RT	14	600/1620	NA	[211]
**Glass/Si/MOF-Based Gas Sensors**							
Soda-lime glass	CO_2_	4000	350	0.4	NA	NA	[156]
Pd@Soda-lime glass	CO_2_	100	350	8.160	NA	0.2	[157]
Si NWs	H_2_	50	100	17.1	505/150	10	[163]
Si/SnO_2_ NWs	H_2_S	50	100	3.5	NA	NA	[167]
Pd@Si nanohorns	H_2_	10	400	~2300	NA	0.1	[168]
Graphene/Si NWs	H_2_	NA	25	1280%	12/0.15	NA	[212]
Pd@p-Si	H_2_S	3	RT	~88%	NA	0.3	[213]
Pd@Si NWs	H_2_	20000	RT	~300%	NA	5	[214]
ZnO@ZIF-8 MOFs	H_2_	50	300	1.44	NA	NA	[174]
SIM-1 nanomembrane@ZnO NWs	H_2_	50	300	~2.5	NA	NA	[175]
Pd@ZnO NWs	H_2_	50	200	6.7	NA	NA	[178]
Ni-MOFs	CO	50	200	1.7	NA	1	[180]
Mg-MOFs	NO_2_	50	200	~1.35	167/92	1	[181]
MOF derived ZnO-CuO	H_2_S	10	350	10.99	58/273	1	[182]
n-ZnO/p-Co_3_O_4_ derived from MOFs	C_2_H_5_OH	10	300	34.9	57/235	1	[183]
Pd@Zr-MOFs	H_2_	100	150	1.94	NA	10	[215]
Pd NWs @ZIF-8	H_2_	0.1%	RT	0.7%	30/8	0.6	[216]
ZIF-67 derived WS_2_@carbon composites	NO_2_	1	RT	18%	NA	0.1	[217]
ZIF-67 derived hollow Co_3_O_4_ nanocages	*p*-Xylene	5	225	78.6	NA	0.25	[218]
Pd@ZIF-67 derived PdO@Co_3_O_4_	CH_3_COCH_3_	5	350	2.51	NA	0.1	[219]
MOF derived ZnO-Co_3_O_4_	CH_3_COCH_3_	5	450	29	NA	1	[220]
TiO_2_-SnO_2/_MWCNTs@Cu-BTC	NH_3_	10	RT	0.58	80/15	0.77	[221]
Cu_3_(HHTP)_2_/Fe_2_O_3_	NO_2_	5	20	89.4%	NA	0.2	[222]

## Data Availability

Not applicable.
